# Micro and Nanostructured Materials for the Development of Optical Fibre Sensors

**DOI:** 10.3390/s17102312

**Published:** 2017-10-11

**Authors:** Cesar Elosua, Francisco Javier Arregui, Ignacio Del Villar, Carlos Ruiz-Zamarreño, Jesus M. Corres, Candido Bariain, Javier Goicoechea, Miguel Hernaez, Pedro J. Rivero, Abian B. Socorro, Aitor Urrutia, Pedro Sanchez, Pablo Zubiate, Diego Lopez-Torres, Nerea De Acha, Joaquin Ascorbe, Aritz Ozcariz, Ignacio R. Matias

**Affiliations:** 1 Department of Electric and Electronic Engineering, Public University of Navarre, E-31006 Pamplona, Spain; parregui@unavarra.es (F.J.A.); ignacio.delvillar@unavarra.es (I.D.V.); carlos.ruiz@unavarra.es (C.R.-Z.); jmcorres@unavarra.es (J.M.C.); cba@unavarra.es (C.B.); javier.goico@unavarra.es (J.G.); miguel.hernaez@unavarra.es (M.H.); pedrojose.rivero@unavarra.es (P.J.R.); ab.socorro@unavarra.es (A.B.S.); aitor.urrutia@unavarra.es (A.U.); pedro.sanchez@unavarra.es (P.S.); pablo.zubiate@unavarra.es (P.Z.); diego.lopez@unavarra.es (D.L.-T.); nerea.deacha@unavarra.es (N.D.A.); joaquin.ascorbe@unavarra.es (J.A.); aritz.ozcariz@unavarra.es (A.O.); natxo@unavarra.es (I.R.M.); 2 Institute of Smart Cities (ISC), Public University of Navarre, E-31006 Pamplona, Spain

**Keywords:** optical fibre sensor, nanotechnology, nanostructured materials, chemical sensing, bio medical sensing

## Abstract

The measurement of chemical and biomedical parameters can take advantage of the features exclusively offered by optical fibre: passive nature, electromagnetic immunity and chemical stability are some of the most relevant ones. The small dimensions of the fibre generally require that the sensing material be loaded into a supporting matrix whose morphology is adjusted at a nanometric scale. Thanks to the advances in nanotechnology new deposition methods have been developed: they allow reagents from different chemical nature to be embedded into films with a thickness always below a few microns that also show a relevant aspect ratio to ensure a high transduction interface. This review reveals some of the main techniques that are currently been employed to develop this kind of sensors, describing in detail both the resulting supporting matrices as well as the sensing materials used. The main objective is to offer a general view of the state of the art to expose the main challenges and chances that this technology is facing currently.

## 1. Introduction

It is well known that optical fibre technology produced a revolution in the telecommunications field during the last decades of the last century. Nowadays, this waveguide provides many homes with lots of services at a high speed. Some of the most relevant features of optical fibre are its high bandwidth, low transmission losses and the possibility of multiplex information. But optical fibre is being employed not only for communications, but also for different applications such the development of endoscopes for medicine thanks to the possibility of carrying light through a probe as well as collecting images by it [1]. It was also thought that the sensing technology could take advantage of optical fibre’s features and replace electronic sensors in many applications; however, electronic sensors were developed and commercialized decades before the use of optical fibre was extended, so that their prices are much more competitive; moreover, the integration of electronic circuits and devices make them very appealing and easy to manipulate. Therefore, it is necessary to find the target applications where the features that optical fibre offers make a difference compared to the electronic counterpart. 

Thanks to its low losses, optical fibre allows remote sensing which is a good property when preparing a multi sensor network; moreover, it is made of an inert material, silica, making it immune to electromagnetic interferences; no electrical biasing is required to guide light, so that the resulting sensors are passive, which is very relevant in environments with an explosion risk. Finally, researchers are developing modulation techniques that allows physical quantities to be measured along the fibre itself, which is called distributed sensing. An application where all these features are required is structural health monitoring in civil engineering [2]: tunnels, bridges, concrete constructions are currently being monitored using optical fibre sensors. The parameters that are commonly registered in these cases are mechanical vibrations, temperature and strain. 

Following with the idea of sensing, the question that arises is if it is also possible to measure chemical entities: to achieve it, a material sensitive to the parameter to be detected has to be deposited on the fibre. In this manner, the guidance of the light is altered by this compound, so that if its optical properties change, they will modify the photonic signal traveling through the fibre, which constitutes the transduction principle of optical fibre sensors for chemical measurements. There is a wide variety of applications were this type of devices can be used: bio sensing [3], pH measuring [4], detection of Volatile Organic Compounds (VOCs) [5], monitoring of relative humidity [6] or gas sensing [7], just to mention some important categories. In most of the schemes, the sensing compound has to be embedded into a supporting matrix, which is almost as important as the sensitive material itself. On one hand, the coating has to keep the reagent attached onto the fibre and, therefore, be robust to chemical attacks as well as to aging; on the other hand, it must allow the target molecules and the sensing material to interact between them, so the transduction can take place (in some cases, the supporting matrix itself is sensitive to the target parameter). As it can be inferred, the morphology of the coating plays a relevant role, and it should be adjusted at a micro- or even nanometric level: in other words, micro and nanostructured materials have to be used.

The development of nanotechnology has allowed deposition techniques to be adapted to get coatings whose morphological parameters can be modulated: volume/surface ratio, porosity, porous size or chemical adsorption are some examples. Of course, the final thickness of the coating is very relevant: in the case of optical fibre, is has to be below a few microns because otherwise, the transduction will not reach the light traveling along the fibre. To be able to handle with these requirements, it is almost compulsory to use nanotechnology: actually, many efforts are being focused to implement of bio and chemical optical fibre sensors thanks to the synergy between this substrate and nanostructured materials.

This review has been prepared to be accessible to readers that are familiarized with optical fibre technology and the ones that are not, as well as for researchers that handle optical fibre but are not used to working with chemical reagents or sensing materials. Firstly, the fundamental concepts of this kind of sensors are briefly presented to understand how they work as well as the transduction principles of the devices described along the section. Thereafter, some construction techniques to develop chemical and biosensors with optical fibres are described: all of them are referenced at the next section where sensors are classified depending on the micro- or nanostructured material used to implement them. This review does not intend to include all the techniques reported in the literature but only some of the most significant. The manuscript review ends with a summary of the concepts, techniques and results exposed; some conclusions about the current state of the art are also presented.

## 2. Fundamentals of Optical Fibre Sensors

This section describes briefly the main concepts about optical fibre sensors to understand the mechanism behind their operation. Thereafter, some of the most relevant transduction principles are described. A detailed study about optical fibre sensors theory can be found in [8].

### 2.1. Theoretical Background and Main Architectures 

**Figure 1:**
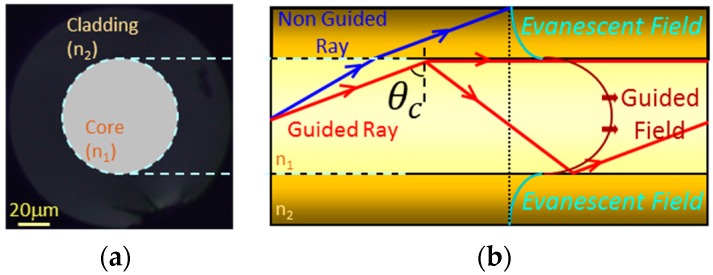
(**a**) On the left hand side, the transversal section of the optical fibre showing core (highlighted) and cladding; (**b**) on the right hand side, the longitudinal section showing the signals involved in light propagation explained by ray theory (arrows) and electromagnetic field theories.

An optical fibre is a waveguide that transmits light of different wavelengths. The propagation mechanism can be analysed by Maxwell equations as well as by ray theory because the signal wavelength is smaller than the physical dimensions of the waveguide. A standard optical fibre is made of silica and, more specifically, has two main parts: core and cladding. The core is typically doped to obtain a refractive index slightly higher than the cladding’s one, which guarantees total reflection by Snell law under certain conditions; however, there is always a small energy transmission from the core to the cladding. Talking in terms of electromagnetic fields, most of the signal is transmitted by core modes, although a tiny part of it is coupled into the cladding modes, also known as evanescent field (see Figure 1). In the case of sensors, the transduction can take place with the core mode or with the cladding ones depending on the architecture and the sensing material employed.

**Figure 2:**
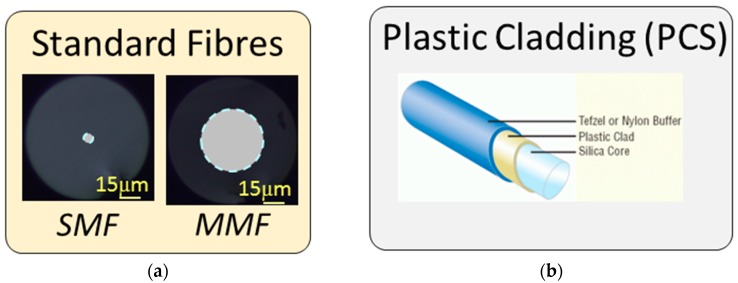

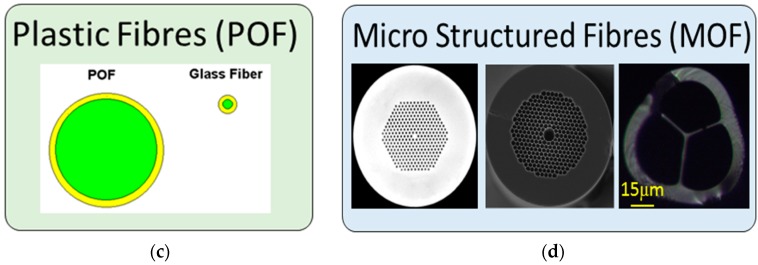
Diagram showing the type of fibres that are commonly used to prepare sensors.

There are several ways to classify optical fibres. The ones used in telecommunication systems are considered as standard by International Telecommunication Union (ITU) and show a cladding diameter of 125 microns. Depending on the core diameter, there are two types of standard fibres: Single Mode Standard Fibre (SMF) and Multi-Mode Standard Fibre (MMF); for the first one, the core diameter below 9 microns, whereas for the second is larger than 50 microns. In both cases, core and cladding are made of SiO_2_, but not all the fibres are made of this material: some of them have a silica core but a plastic cladding, and are known as Plastic Cladding Silica (PCS) core fibres. This kind of fibres is typically wider, with core diameters ranging from 100 microns up to 1100 microns. Moreover, the plastic cladding is easy to remove, which easies the interaction with the evanescent field. Another type of fibre is completely made of plastic, which is named Plastic or Polymer Optical Fibres (POF): they are commonly wider as SMF or MMF, and have different applications in communications, sensors and in the biomedical field due to the mechanical robustness, the improvement of their transmission properties and the easiness to manipulate them [9,10,11]. Finally, there are fibres that are fabricated with air holes following a pattern: Microstructured Optical Fibres (MOF). Of course, several subgroups can be made in this category, but they will be detailed further in this paper considering these fibres as microstructured materials. There are other types of fibres, but these are the common ones used for sensors. Figure 2 shows a brief summary.

**Figure 3:**
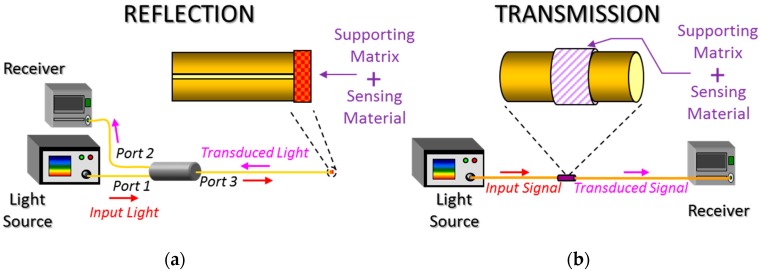
Architectures for intrinsic optical fibre sensors: (**a**) reflection and (**b**) transmission.

In an optical fibre sensor, the transduction takes place when the target analyte changes the properties of the light traveling through the waveguide. If the transduction occurs externally to the optical fibre, the sensor is extrinsic, whereas if it occurs on the fibre it is named intrinsic by some authors [5]: most of the sensors studied for this review are intrinsic. Depending on the location of the sensing layer, two types of architectures or configurations are considered: transmission and reflection (see Figure 3). In the case of reflection sensors, the sensing material embedded into a supporting matrix is deposited at the end of a pigtail (which can be perpendicularly ended or show other shapes); sensors prepared with this configuration are known optrodes because they look like electrodes. The key device for a reflection set up is the optical coupler which guides the signal from the light source to the sensor, and from it to receiver. They are mechanically robust and easy to manipulate, although the reduced interface yields to low signal levels. On the contrary, for transmission sensors there is a direct optical path between the light source and the receiver, so that the optical losses are lower compared to the other configuration. Furthermore, the interaction with the sensing material can be enhanced by stretching the fibre or removing its cladding. However, the resulting sensor is mechanically weaker and has to be attach to a holder to prevent its breaking. To choose between them, the final application requirements, the transduction principle and the sensing material have to be taken into consideration. 

### 2.2. Optical Absorption

**Figure 4:**
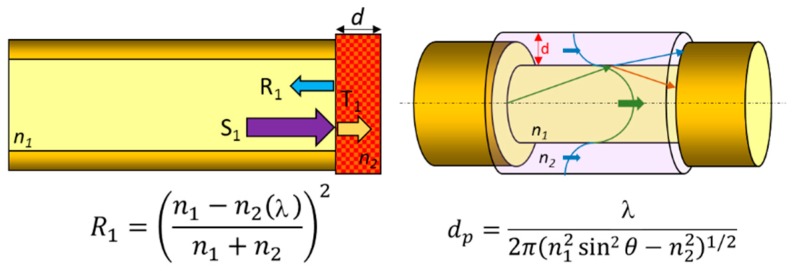
Scheme of the optical absorption phenomenon for a reflection sensor (on the **left**) and a transmission one (on the **right**); below each one, the Fresnel equation for the reflected power and the expression for the penetration depth of the evanescent field.

This transduction principle is based on the changes of the optical properties of the sensing material deposited onto the fibre. In the case of reflection sensors, the intensity of the reflected signal is described by the Fresnel equation, which depends on the refractive index of the sensing coating; if this parameter is alter by the entity to be measure, the reflected signal will be different. Moreover, as the refractive index of the material may depend on the signal wavelength, if a white light source is used to illuminate the sensor, then the colour of the film is reflected and it is possible characterize the whole absorbance spectra of the material: colorimetry measurements can be done this way. Regarding to transmission sensors, the evanescent field can be modified by changes of the sensing coating refractive index, which increases or decreases the light coupled to the cladding modes. A critical factor is the penetration depth of the evanescent wave, which depends on both the refractive index of the core and the coating one. Figure 4 displays and scheme reflection and transmission configurations for this transduction mechanism. The interaction with the evanescent field can be increased by stretching the fibre: this kind of devices are known as tapers. Just to have a general idea, the more the fibre is stretched, the more modes are coupled into the evanescent filed, although there is a trade-off with the mechanical robustness of the resulting stretched segment [12,13,14]. Other propagation parameters, such as the optical signal polarization, have been used for chemical sensing [15].

### 2.3. Luminescence

**Figure 5:**
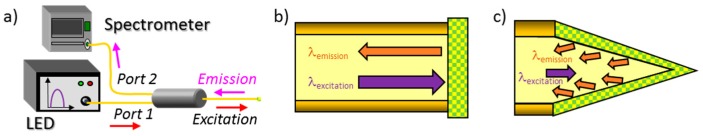
(**a**) Reflection set up for a luminescent sensor; (**b**) perpendicularly ended fibre and (**c**) a stretched one to get more luminescence emission coupled into the fibre.

There are luminescent materials whose emission is reversely quenched by the presence of the analyte to measure. To develop sensors, optical fibre can guide the exciting signal towards the supporting matrix loaded with the luminescent compound as well as it couples back the emission signal from the material. The architecture commonly used for luminescence sensors is the reflection one. In order to get a wider interface, fibres are sometimes stretched to get a cone shape. Figure 5 shows the experimental set up used for this transduction and the signals involved together with two common fibre ends.

### 2.4. Interferometry

**Figure 6:**
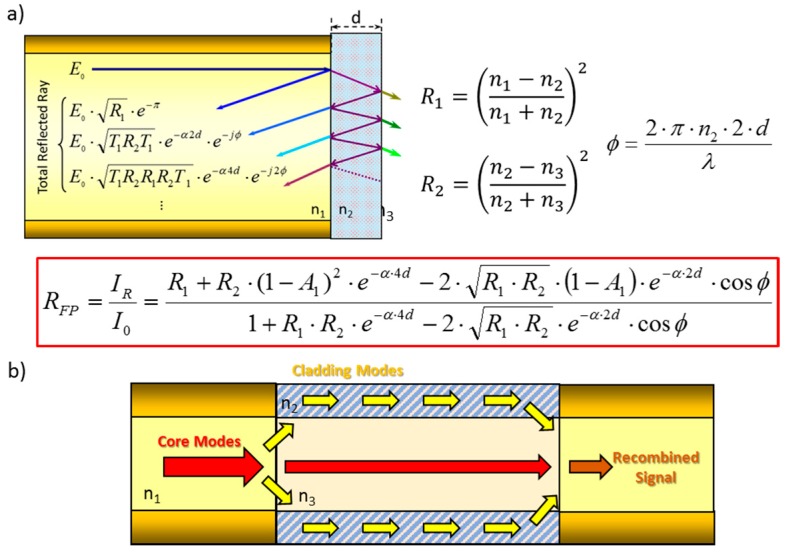
Interferometers used for optical fibre sensors: (**a**) Fabry Perot in reflection sensors, and (**b**) Mach-Zehnder for transmission configuration.

For reflection sensors, interferometry is produced by the interface between the fibre and the sensing coating, and the one formed by the sensing coating and the environment (see Figure 6a). Each one of these interfaces produces a reflection, and both interfere when they reach the fibre core: this nano structure is a type of Fabry Perot (FP) interferometer (more information about optical fibre FP configurations is available in [16]). There are some factors that determine if the interference is destructive or constructive, but the most relevant one, is the thickness of the nanocavity deposited at the end of the fibre: in some cases, it can be increased by the presence of the target analyte, which produces a change in the resulting from the interference mentioned above. Other parameters that determine the interferometric response are the refractive index of the coating and its optical absorbance. The detailed formula is displayed in Figure 6a. A different interference takes place for transmission sensors: when a distinct type of fibre is spliced between two segments of a similar one (for example, a SMF—MOF—SMF section), core modes are redistributed when they reach the first interface to the cladding ones that travel through the sensing coating at different conditions. This effect produces a phase shift between the core modes and the cladding ones so that, when the last reach the second interface and all of them are recombined, an interference occurs. The length of the section of the fibre that is different as well as the thickness and refractive index of the sensing coating, determine this interference: the last parameters are altered by the analyte to be detected, which produces the transduction. Figure 6b shows a diagram of the interfering signals present in this configuration, which is a representative design of a Mach-Zehnder interferometer.

### 2.5. Electromagnetic Resonances

There are different types of resonances that can be induced in optical fibre: in most cases, a specific wavelength is not forward transmitted but reflected or coupled to the cladding depending on the physical phenomena. The presence of a sensing material shifts this wavelength when it reacts with the variable to be detected, which constitutes the transduction mechanism.

**Figure 7:**
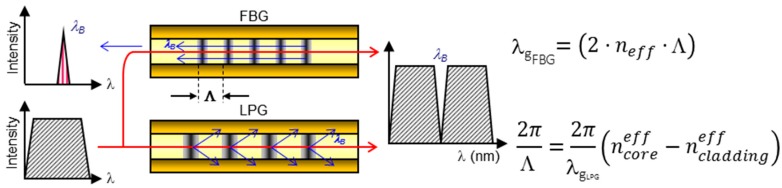
Working mechanism from a FBG and a LPG: inset, the formula for each case that determines the Bragg wavelength.

Fibre Gratings are devices that have a periodic perturbance of the core refractive index along a certain segment. A key parameter of a grating is the pitch of variation: for values in the nanometric scale, a certain wavelength is reflected, whereas for a period in the micrometric range, the wavelength is coupled to cladding modes. The first type of devices is named Fibre Brag Gratings (FBGs) [17] and the second one Long Period fibre Bragg Gratins (LPGs) [18]. The wavelength of interest (known as Bragg wavelength λ_g_) in each case can be calculated by formula that are shown in Figure 7. The dependence on the pitch have make them a good choice for measuring temperature or mechanical strain. Some chemical reagents can also produce a strain by a swelling effect, which can be used for chemical or biological sensing. Furthermore, another way to modify λ_g_ consists of depositing sensing materials that change the effective refractive index of the cladding. There are several approaches that handle with this kind of mechanism for both LPG and FBG, and they will be presented in Section 4. Other structures based on fibre gratings such as Tilted Fibre Gratings, have been also used for bio- sensing [19,20].

**Figure 8:**
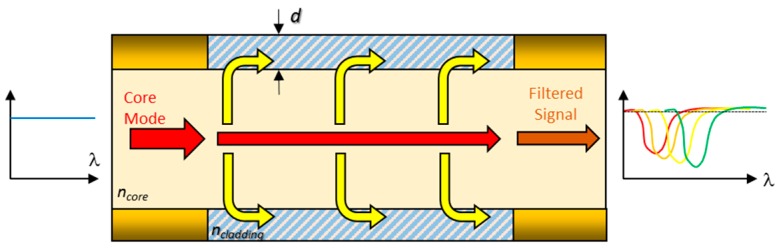
Schematic representation of a LMR: the optical resonance expressed as a transmission valley is increased and shifted if the thickness of the coating or its refractive index is modified.

Another kind of resonance is obtained when the cladding is removed and a coating is directly deposited onto the core. Some metals such as gold or silver induce Surface Plasmon Resonances (SPR) that can be used for sensing. This kind of nano-coatings force a certain wavelength to be coupled into the coating and depend on the metal as well as the refractive index of the environment surrounding it [21]; however, the versatility of SPR is restricted to metallic coatings. On the contrary, a coupling to cladding modes can be also obtained by coatings made of metal oxides and even polymers: they are known as Lossy Mode Resonances (LMRs). These resonances appear for a certain coating thickness as well as for a specific refractive index value of the film: if any of these parameters is changed, the spectral location of the resonance is shifted, which can be used to characterize a sensor. LMRs have been reported relatively recently and show a great potential for sensing applications due to the wide range of materials that induce them [22]. Figure 8 summarizes the mechanism of this transduction principle.

## 3. Techniques for the Development of Sensors

There are several manners to deposit sensing coatings onto optical fibre. The small dimensions of the fibre is one of its advantages, but it also makes difficult coating films that have to be as uniform as possible. Therefore, different approaches have been adapted to handle with this substrate. In any case, a high reproducibility is compulsory to validate the sensor construction process. The most used procedures are briefly described along this section: there are other such as functionalization by inorganic and even biological molecules that can be found in [23,24,25,26].

### 3.1. Dip Coating

**Figure 9:**
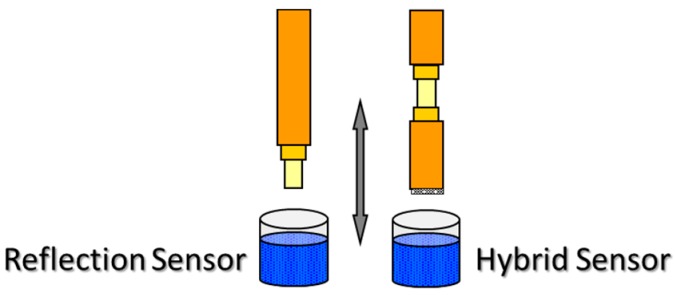
Sensing configurations applicable for dip coating.

This method is one of the easiest to perform: it just consists of dipping the fibre vertically into the solution or dispersion where the sensing material is dissolved or dispersed and then, the fibre is removed from the solution (see Figure 9). The key parameter is the withdrawal speed: the fastest it is, the thicker is the resulting coating. Other important factor is the viscosity of the solution and also a post curing to ensure that any solvent remains are evaporated. The process can be repeated as many times as required to get the desired thickness. As the fibre is immersed perpendicularly, this procedure is only applicable for reflection configuration or a hybrid one. Moreover, there is no control on the film thickness in the case the material is to be coated at the end of the fibre; actually, the shape of the deposition looks as a matchstick. 

Dip coating is typically used for the deposition of sol gel and plastic matrices. Furthermore, it is commonly the first approach employed to test a sensing material or supporting layer and, thereafter, their deposition is optimized following other methods. There are some variations for transmission sensors, for instance, drop casting [27,28]: a drop of the solution is hold at the exit of a needle, and then, the drop is moved along the segment with no coating. Curing is necessary to eliminate the solvent remains, and the process can be repeated as many times as it is required. 

### 3.2. Layer-By-Layer Nano Assembly (LbL)

**Figure 10:**
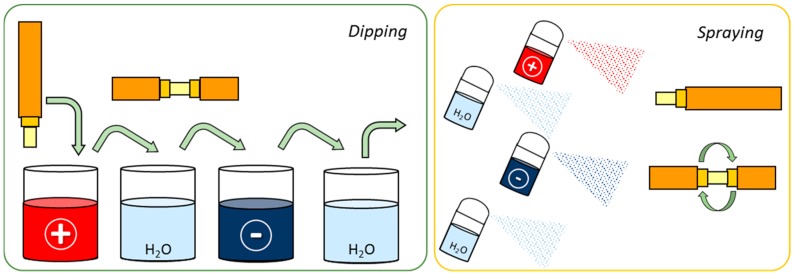
LbL nanoassembly applied for optical fibre (reflection or transmission configurations) by dipping (image on the left hand side) and by spraying (image on the right hand side).

The dimensions of optical fibre make challenging to deposit coatings and to control their morphology at a nanometric level. However, LbL technique fits these requirements. It is based on the assembly of polyelectrolyte chains that show different electrical charge by electrostatic and other weak forces (such as Van der Waals ones). The method was briefly presented in the 60s just for microparticles [29], but it was not until the 90s where the full potential of this technique for a wide range of polyelectrolytes was exposed by Decher [30]; firstly it was proposed for flat substrates, but it can be applied to surfaces of different geometries and dimensions, which is the case of optical fibre. Just in a few words, the substrate is to be immersed into poly cationic/anionic solutions alternatively for a certain time to let the chains get assembled; in between, rinsing is necessary to remove the not properly assembled molecules. The final morphology depends on the number of immersions, the solutions pH, the polyelectrolytes concentration, ionic strength, among other factors that are to be optimized to get the target morphology. The process is easily automated, which makes the sensor implementation repetitive. It can be used for both reflection and transmission architectures; moreover, the solutions can be sprayed onto the substrates (spray coating), which produces thinner layers [31] (see Figure 10 for more details): for transmission configuration, rotation is required to ensure a homogeneous coating around the fibre, whereas for the reflection scheme, as the solutions are perpendicularly sprayed onto the end of the fibre, it is not necessary. 

**Figure 11:**
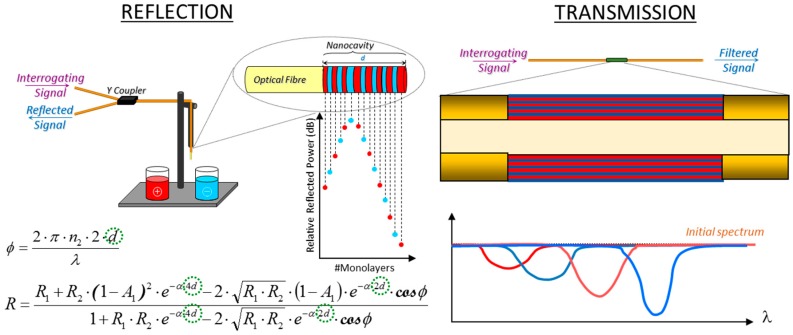
Mechanisms to control the nanocoatings growing for reflection and transmissions configurations: the first one is based on interferometry whereas the second on LMRs.

It is also important to highlight that the LbL growing can be monitored on real time. In the case of reflection configuration, the nanocating that is growing at the end of the fibre forms a FP interferometer in a way its interferometric response depends critically on its thickness: applying the mathematical expression of the FP interference, it is possible to estimate the final width of the coating. On the other hand, depending on the polymers used, LMRs are induced when LbL is used for the transmission configuration: registering the spectral shift produced by the nanocoating thickness increase permits its growing to be controlled. Any of these methods allows the deposition process to be monitored, being a way to confirm the repeatability between different devices, as it has been depicted in Figure 11.

### 3.3. Sputtering

**Figure 12:**
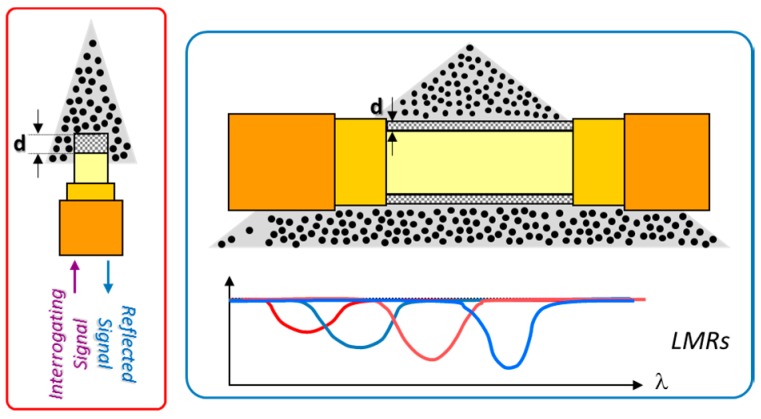
Sputtering deposition for a reflection sensor (on the **left**) and for a transmission one (on the **right**).

Metallic materials are easy to deposit by physical vapor deposition or sputtering. This method uses a target of the compound to be deposited and it is located in a chamber at a high vacuum conditions: thereafter, an inert gas under a strong field generates plasma; these gas ions also act as energetic particles that bombard the solid target provoking the ejection of particles from the target. The resulting coating is uniform, and its morphology depends on the sputtering time mainly, as well as the vacuum level and the signal used to generate the electrical field. DC and AC signals are employed to deposit metals and metal oxides, but currently there are available devices working with Radio Frequency signals that allow a wider variety of materials to be deposited. In the case of optical fibre, it has been found a high reproducibility when preparing the sensors. In this manner, it can be used for both reflection and transmission configurations (see Figure 12): for the first one, the growing of the nanocoating can be monitored by the reflected signal (FP interferometry), whereas for the second one, by registering optical resonances (LMRs) or interferometric shifts produced by the increase thickness of the deposition (for instance, SMF-MOF-SMF Mach-Zehnder). 

### 3.4. Electrospun Nano Webs

It is well known that the final sensitivity of the device depends on the interaction between the target material to detect with the sensing compound, and therefore, the morphology of the supporting matrix. Looking for a high surface/volume ratio rate, porous substrates are a goal for researchers: in this context, electrospinning allows fibres with diameters between 10 and 1000 nm to be deposited, and what it is more interesting, conforming a web-like deposition with just a continuous fibre. This technique was first patented at the beginning of the 20th century and it has been studied and improved since them [32]: actually, it was at the end of the 90 s when it was thought that it showed a great potential for sensing applications. Nowadays, it is possible to get an interaction area up to 1000 times larger when compared to a compact coating.

**Figure 13:**
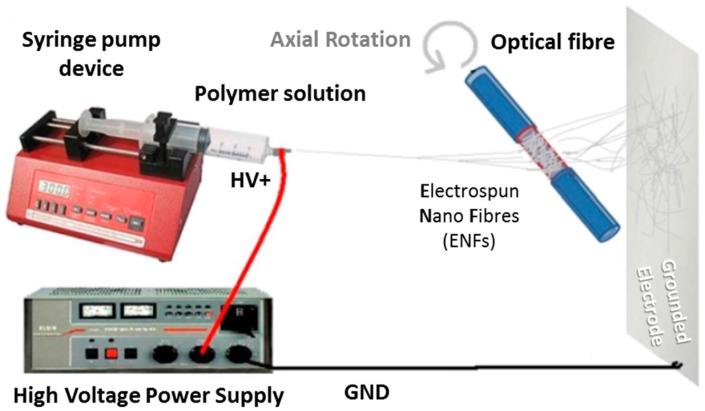
Set up for an electrospinning deposition onto an optical fibre. The main relevant elements are the syringe pump device, the high voltage source and the grounded electrode. Reprinted from [37] with permission from Elsevier.

Electrospinning is based on the stretching of a viscoelastic solution produced by electrostatic forces: the material to be deposited is dissolved and the solvent employed is evaporated during the deposition process. Initially, the procedure was mostly performed with polymers and organic solvents [33], although recent studies report that materials such as metal oxides [34,35] or porphyrines [36] have been successfully deposited. For a conventional set-up (see Figure 13), the solution is injected into a syringe and then, a critical voltage (between 5 and 15 kV) is applied between a needle attach to it and a ground electrode. The solution gets electrically charged in a way that the electrostatic repulsion overtakes the cohesion forces, breaking the superficial tension: as a consequence, a jet emerges from the solution and literally it flights towards the grounded target. As the jet travels, the solvent gets evaporated, getting thinner as well and eventually bended, describing a helix path. The final deposition is similar to a non-woven fabric with a high porosity, whose fibres alignment depend on the parameters mentions above. Finally, the nanoweb is dried under different conditions (vacuum, nitrogen, etc.) at a certain temperature to remove any remaining solvent.

There are many parameters that determine the morphology of the nanofibre and also the one of the resulting nanoweb attach onto the substrate: for instance, if the solution viscosity is high enough, a continuous jet would be ejected, whereas if it is low, droplets would be obtained instead. Other relevant factors are the solution conductivity, the applied electric tension (also if it is alternating or direct current), the distance between the electrodes, the syringe flow rate and even the relative humidity. The type of grounded electrode also plays a relevant role: circular rotative discs or an array of counter electrodes produce different alignments of the jet once it is deposited on the substrate. In the case of the nanoweb is to be coated onto optical fibre, the substrate has to be rotating during the process (as it is displayed in Figure 13): rotation speed of the fibre is a relevant factor that determines the morphology of the final supporting matrix. 

## 4. Nano and Microstructured Materials for Sensing

This is the main section of the review. Relevant results for optical fibre sensors will be presented considering the nano- and microstructured materials used to implement them. All the sub sections are based on the sensors reported in the bibliography along, approximately, the last 17 years. Apart of the materials that are to be presented, there are others that, due to their extended used in different approaches, are difficult to classify individually: it is the case of nanoparticles (NPs) that are employed to enhance the sensitivity of optical fibre sensors of different architectures and transduction principles. Some of the most used NPs are gold [38] and silver [39], and it has been found that even the shape of the NP should be considered to get optimized results [40]. More details about the use of NPs to develop optical fibre sensors can be found in [41].

### 4.1. Microstructured Optical Fibres 

**Figure 14:**
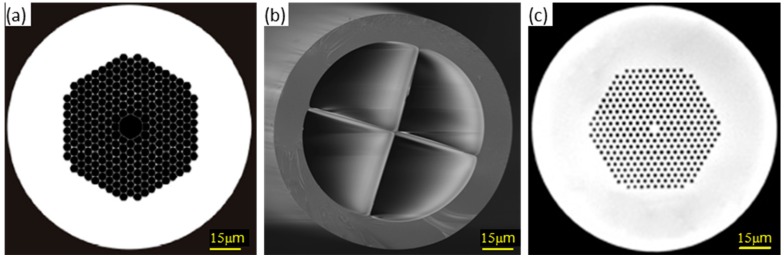
SEM images for (**a**) a Hollow Core fibre, (**b**) a Suspended Core fibre and (**c**) a Photonic Crystal one. Image (**a**) is reprinted from [55] with permission from Elsevier.

The optical fibre itself can be modified or designed at a microscale level, so in certain cases, it could be considered as a microstructured material. For standard fibres, light transmission is possible because of the refractive index difference between the core and the cladding. However, a new type of fibres has been developed in which the propagation is produced by the geometrical distribution of air holes that make the light transmission possible. The resulting structure is in the micro scale, so that this type of fibres is named Microstructured Optical Fibres (MOFs) [42,43,44]. There are several distributions for the air holes: some of them confine the light through the fibre core, which is employed for high power signal guiding; on the contrary, other distributions force core modes to be coupled into the cladding ones, which is very useful for sensing applications [45]. There are three main types of MOFs: Hollow Core Fibres (HCF), Suspended Core Fibres (SCF) and Photonic Crystal Fibres (PFC), which are displayed in Figure 14. The distribution of the air holes as well as their dimensions determine the transmission properties of the fibre, so that there are tens of subtypes in each category. Furthermore, the design of the MOFs can include periodical modifications that yield to optical fibre gratings [46,47]: the holes can be functionalized with the sensing material, so that in presence of parameter to measure, the interaction between them shifts the Bragg wavelength [48,49,50]. 

The air holes make MOFs an option to be considered for liquid and gas sensing [51]. Manufacturing this kind of fibres is getting cheaper and there are many different types commercially available. There are also some drawbacks such the influence of ambient pressure: to overcome it, designs are under study and they are currently in a development stage [52]. Other fabrication approaches include the cladding functionalization to attach sensing coatings more easily [53], while ion beam technique is also used to create microfluid channels on the fibre [54].

In the case of HCF, they are commonly used for high intensity signals because of their nonlinear coefficient is smaller than the standard fibre’s one. Regarding SCF, the core is hold by tiny silica bridges that make de impression of a hanging one: the narrower it is, the stronger the evanescent field in the air holes is. This type of fibre is very sensitive to changes in the properties of the air holes. Actually, efforts are focused on the development of SCF with cores as small as possible to enhance this effect [56]. Sensing materials can be deposited on the walls of the holes, providing to the sensor the required sensitivity: depending on the chemical reagent, the transduction is governed by changes in the refractive index and/or in its optical absorbance. Metal nanoparticles have been fixed by microfluidic methods in order to obtain very high sensitive refractometers [57] and even sputtering can be applied to deposited metallic oxides. HCF is currently showing a great potential for biomedical sensing: the large air holes can be used as micro fluidic cavities to detect target molecules such as proteins or pathological agents (sensing material is previously deposited on the inner walls), so that only micro or even nanolitres are required to make the measurement [58]. Some authors also proposed that HCF properly functionalized could be used for distributed sensing in hydrogen leakages detection [55].

**Figure 15:**
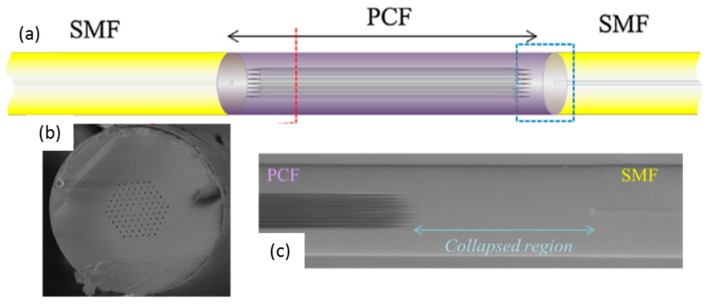
(**a**) Scheme of an interferometer based on a PFC segment spliced between two SMF; (**b**) SEM image of the PFC section; (**c**) optical microscope capture of the interferometer, delimiting the three different parts. Reprinted from [60] with the permission from Elsevier.

PCF are named this way because the patterned air holes distribution is similar to a crystal structure (some authors sub divide PCF into solid and hollow core groups): the solid core is surrounded by an area of lower refractive index due to the air holes, which enhances the coupling to cladding modes. Taking advantage of this effect, a sensing layer can be deposited onto a PCF section with an optimized thickness (below 1–2 microns approximately) that ensures the interaction with the target analyte and the evanescent wave. This kind of fibre is used to prepare interferometers by splicing a PCF between two standard fibres (see Figure 15), which generates an interfering spectral pattern that is shifted by the presence of the entity to measure. Sensors have been developed this way to measure relative humidity [59,60] and the architecture can be extended to other applications depending on the sensing material.

### 4.2. Nanostructures

**Figure 16:**
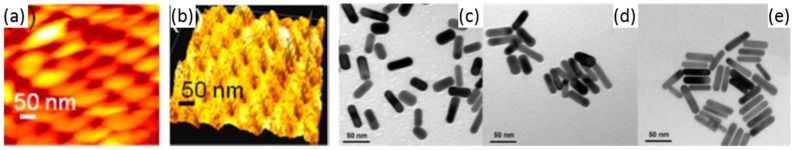
(**a**,**b**) show the nanoporous structure observed from a hybrid polymer—metallic structure; (**c**–**e**) images on the right hand side display gold nanorods with aspect ratios to detect immunoglobulins. Reprinted from [62,63] with permission from Elsevier.

Advances in nanotechnology have allowed structures made of different materials to be constructed in the sensor field. Properties such as sensitivity to refractive index changes, large interaction area or periodic patterning are typically required. Materials that are relatively easy to deposit are metals by sputtering or CVD, which produce plasmonic resonances; on the contrary, non-conducting materials produce photonic resonances: both of them can be combined to design structures at a nanometric scale, which are known as hybrid structures [61]. This kind of constructions can be functionalized to detect biological molecules such immunoglobulins taking advantage of the nanoporous morphology that this type of material offers: as it is reported in [62], a polymer layer can act as substrate (for instance, polydimethylsiloxane), and on it, a column based nanostructure prepared with anodic aluminium is deposited; thereafter, the coating of a nano-Au layer yields to a porous surface that is finally functionalized with protein receptors (Figure 16a,b). As a result, variations in the absorption spectra are recorded when the structure is exposed to biological solutions containing the target protein. Gold nanorods (Figure 16c–e) can be also synthetized to detect human immunoglobulins in terms of spectral shifts [63]; gold nanorings have been also been synthetized to work on the optical fibre operating spectral range to prepare a potential refractometer [64]. The transduction principle of these materials is based on spectral shifts.

**Figure 17:**
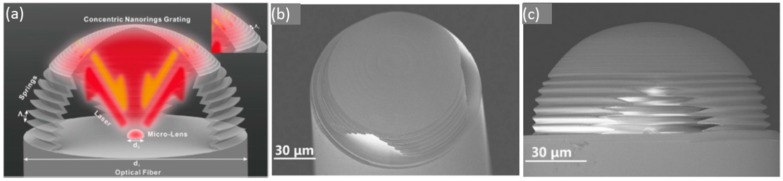
(**a**) 3D design of the microcavity with the micro lenses to implement the optical fibre microphone; (**b**) detailed of the top view of the cavity and (**c**) its side view, where the lens can be seen. Images reprinted from [67] with permission of Elsevier.

Some structures have been successfully constructed onto the optical fibre. By using different masks to deposit the sensing material, cavities or resonators can be used as transducers. Thanks to its plasmonic resonances, gold has been used to prepare concentric circles on the top end of a fibre, in a way the resulting probe is highly sensitive to changes in the external refractive index [65]. Non-conducting materials, such as composites, are also employed to construct microcavites that enhance the interaction between the chemical target to be measured and the optical signal: a microprism has been designed to implement an interferometer at the end of a standard optical fibre to detect chlorinated organic solvents [66], Not only chemical analytes are to be measured by using nanostructures: acoustic waves can also be registered by an optical fibre microphone [67]. The transduction principle of this sensor is based on a microcavity made of IPL-780 photoresist (Nanoscribe GmbH, Baden-Württemberg, Germany), which is placed at the end of a fibre tip. Microlenses are distributed inside the cavity, so that they reflect the optical signal that reaches them from the fibre towards back (see Figure 17b): in the presence of acoustic waves, the lenses are displaced, and so, the reflected signal varies, which constitutes the sensor working principle. Other nanostructures prepared with photo resins have been reported by following nano imprint lithography [68].

In addition to these structures, the concept of “lab-on-fibre” is an emergent field to consider in the next years. Some examples of the “lab-on-fibre” potential can be found in works from Cusano and co-workers are, among other achievements, a phase-gradient plasmonic metasurface on the fibre tip; the nanosphere lithography of the optical fibre end facet; even a method for integrating sub-wavelength resonant structures on top of optical fibre tip based on direct milling of the glass on the fibre facet by means of Focused Ion Beam (FIB) have been recently reported [69,70,71]. FBI technique is very similar to scanning electronic microscope but employing an ion beam instead of an electron one. In this manner, different shapes and structures can be inscribed onto the fibre and, moreover, different materials can be deposited too. This technique is offering very promising results, so that sensors are supposed to be prepared this way in near future [21].

### 4.3. Plastic Matrices

Mechanical robustness is always required for the sensing coatings as well as a negligible reactivity to aging agents. These are two of the most important features that plastic matrices offer for the development of optical fibre sensors. The plastic is mixed with an organic solvent together with the sensing material and, in some cases, with other substances such as plastifiers: it is compulsory that they do not react with the sensing molecules to preserve their sensitivity. The technique commonly employed to deposit them is dip coating, so that depending on the number of immersions and the dipping speed, coatings with different morphologies are obtained. A final curing stage is required to remove the organic solvent and to harden the polymer, which enhances the mechanical properties of the resulting supporting matrix.

**Figure 18:**
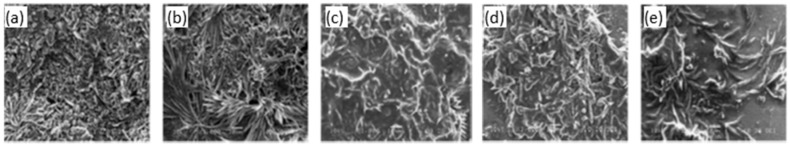
SEM images from cobaltous—polyaniline coatings with different thickness: (**a**) 10.38 μm, (**b**) 17.26 μm, 21.08 μm (**c**), 23.57 μm (**d**), and (**e**) 26.03 μm. It can be appreciated in the first images (a and b) that the morphology is more porous that for the last ones (**d**,**e**). Reprinted from (Vijayan et al., 2008) with permission of Elsevier.

Most of the polymers employed are hydrophobic, which is also a desirable feature in cases such as the detection of gaseous ammonia based on pH indicators: hydrous matrices can get degraded under dry conditions, so that hydrophobic coatings overcome this situation. Bromocresol Purple has been mixed with a polyurethane plastic, Tecoflex^®^, and ethanol, preparing a solution into which a PCS fibre was dipped several times at a certain speed. The resulting sensor, based on the absorbance spectral changes of the chemical indicator produced by ammonia molecules, shown a repetitive and sensitive response to this agent [72]. Other very well-known plastics such as poly (vinyl chloride) (PVC) have been used to prepare sensors that detect pyridine vapours [73] and europium ions dissolved in water [74]: it is important to keep in mind that some polymers are opaque, so that there is a trade-off between the coating thickness and the signal level that determines the final sensitivity. Polymers that yield to high porosity matrices that promote gas adsorption are required, for example, in the case of oxygen sensing: polystyrene (PS) was used to deposit luminescent metallic porphyrines to prepare an oxygen sensor [75]. The coating thickness is critical for most sensors prepared with plastic matrices: if the coating is too thin, there is a low interaction with the evanescent wave, but if it is too high, the transduction might not reach the area where the evanescent field is interacting with the membrane. What is more, the thickness also determines the morphology in terms of porosity. For a cobaltous deposition into a polyaniline matrix to measure humidity [76], it has been noticed that the thinner the layer is, the more porous the morphology is, which improves the sensitivity (see Figure 18).

Not only the plastic membrane acts as a supporting element, but sometimes also as the sensing one: depending on its optical properties (such us the refractive index), some plastics have been employed to implement evanescent wave sensors. Polymers with high refractive index promote the coupling of the cladding modes into the sensing membrane: this effect was taken advantage to enhance the sensitivity of LPG based refractometers [77]. Although for this example the deposition technique was dip coating, the plastic—sensing element batch can be also sprayed onto the fibre to get the thin film: a siloxane polymer film with a thickness of just some nanometres was obtained in this manner to implement a device able to detect benzene, toluene and *o*-xylene [78].

### 4.4. Sol Gel

**Figure 19:**
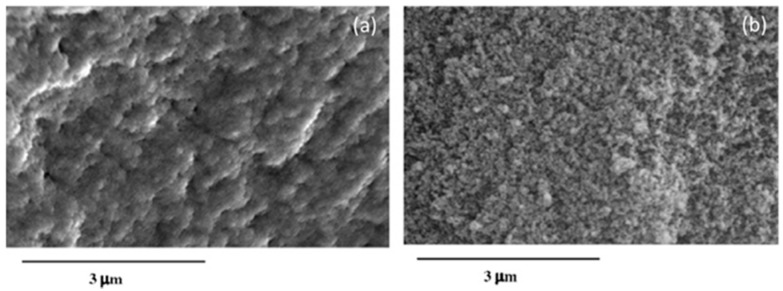
SEM images for a (**a**) xerogel and (**b**) an aerogel. The porous diameters in the first case are between 20 and 40 nm; for the second one, they are above 50 nm. Reprinted from [80] with permission from Elsevier.

The supporting matrices are required to be porous to easy the adsorption of the target analyte: sol gel technique allows the size and morphology of the porous to be controlled. This technique is based on silica (among other organic and inorganic reagents), the same material which optical fibre is made of: not only sol gel matrices show similar refractive index and transmission losses but also it ensures a good adherence to the substrate: therefore, it has been used for optical fibre sensors implementation since the first devices were reported [79]. The synthesis of the coating relies on a polymerization reaction of semi metallic or metallic hydroxides: other chemical reagents present in the process are water, an organic solvent, a precursor and a catalyser. The kinetics of the process as well as the properties of the resulting matrix depend on pH, reagents concentration and more critically, on the curing process: if an atmospheric environment is used, the matrix is called xerogel, whereas if it performed at high temperature and pressure an aerogel is obtained (see Figure 19). The second one shows a higher porosity [80]. To prepare optical fibre sensors, the fibre is dipped into the solution once gelification has started, so that the curing process takes place on it. Thickness and length of the coating are to be optimized to get a proper sensitivity [81].

**Figure 20:**
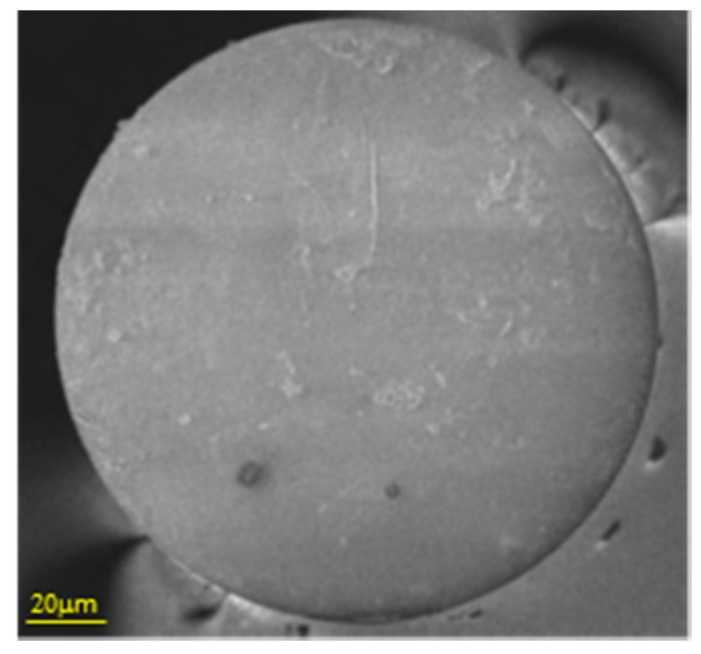
SEM image from a multimode cleaved ended fibre onto which a xerogel has been deposited to measure humidity changes; the uniformity of the resulting coating once the curing process is finished is remarkable. Reprinted from [83] with permission from Elsevier.

In the case of silica based sol gels, the final matrix can act as the sensing element itself with no need of a sensing material. It has been hypothesized that silanol groups might interact with molecules showing lone pairs of electrons, which is the case of some VOCs: as a result, the optical properties of the coating changes and variations in the absorbance spectra are used to detect the VOCs presence [82]; even relative humidity sensors are reported employing just xerogel as the sensing element (see Figure 20) [83]. Sol gels have been also coated onto optical devices such as stretched fibres and micro couplers: the response of this sensors is based on spectral shifts. These configurations work in transmission so that dip coating is difficult to perform: drop coating is used instead. Sensors are reported following this method to detect ammonia gas [84] and ammonia dissolved in water [85].

Sol gels can be also doped with a sensing material, in which situation they also act as the supporting matrix. Depending on the chemical properties of the reagent, it is added at different stages of the solidification process but always when viscosity is low enough. It is critical that the sensing features of the material are preserved and, of course, that it does not interfere the gelification reaction. Commercially available sol gel solutions have been used to implement sensors using chemical dyes to measure pH [86] and organometallic compounds to detect and identify VOCs [87]. The variety of materials that can be deposited is remarkable: sol gel matrices successfully doped with zinc oxide have been used to implement humidity sensors with coatings showing just some tens of nanometers width [88]. Moreover, taking advantage of the optical transparency of this kind of thin films, luminescent materials can be embedded into them, so that the luminescent emission is coupled to the fibre with low losses. Luminescent materials are chemically complex, so their adding to the gelification process has to be optimized as much as possible in terms of sensing material concentration. Lead detection in water has been achieved by using a fluorophore as sensing element into a sol gel matrix [89]; another metal ion, Cu^2+^, has been successfully monitored by an optical fibre sensor prepared with a sol gel matrix doped by metalloporphyrins and quantum dots [90]. Moreover, the high porosity of sol gel coatings makes them optimal for gas sensing, so that different studies report the use porphyrins embedded into this supporting matrix to detect oxygen [91,92,93]. Xerogels can be also doped with organometallic materials to detect VOCs taking advantage of their high porosity [94].

**Figure 21:**
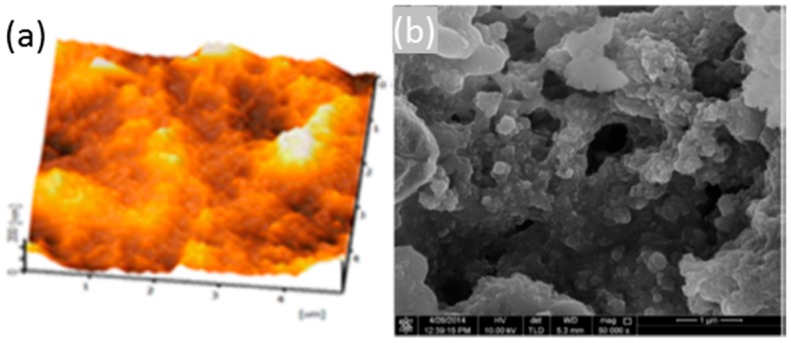
Sol gel coatings prepared with a mixture of SiO_2_ and TiO_2_: (**a**) AFM image of the coating doped with a pH indicator and (**b**) a SEM image to highlight the porous structure of the layer. Reprinted from [95] with permission of Elsevier.

Recent works detail the combination of silica and titania (TiO_2_) to obtain sol gels with a high porosity and mechanical robustness; as before, the matrix can be doped with sensing materials such as pH indicators to implement sensors [95]; other agents, for instance surfactants, are included to increase the coating porosity, in which case thickness is between 2 and 4 microns. Figure 21 displays AFM and SEM images of the thin films with different widths with a noticeable porosity. The ratio between SiO_2_ and TiO_2_ also determines the final refractive index of the sol gel (from 1.5 up to 1.9): this parameter can be optimized when preparing sensors based on LPGs. These devices are very sensitive to refractive index changes in the environment (atmospheric, or liquid), so if the coating is prepared to maximize the coupling from the optical resonance, a high sensitivity can be obtained. Furthermore, the fibre can be dipped into solutions with a sensing material to functionalize the supporting matrix in a way that compound is trapped into the porous layer once the fibre is removed from the solution [96]. As a result, bio sensors concentration have been implemented to measure immunoglobulins in terms of the Bragg wavelength shift produced by the transduction [97]; devices to detect VOCs have been also reported using LPGs coated with sol gel functionalized by calixalane [98]. Other hybrid sol gel compounds that handle with gold nanoparticles are currently be under study for luminescent based sensing applications [99].

### 4.5. Metal Oxides

There are several reasons that explain why metal oxides are a promising material for sensor development: thanks to its nature, they are thermally robust, showing also an optimal mechanical robustness; their properties are not affected by aging and they have a good resistance to chemical degradation [100]. The ones used for sensor development are semiconductors, so that when they react to the target analyte, the electrical conductivity changes. As can be inferred, the first developed sensors based on metal oxides were electronic and variation in the electric resistance was measured; however, the oxidation/reduction reaction that produces conductivity changes required heating the material up to some hundreds of degrees Celsius, which is not feasible for VOC measurement. Therefore, other changes produced by the presence of the reacting agent have been used: luminescent properties or variations in the refractive index are some examples. Another way to prepare sensors by metal oxides consists of coating a thin layer that generates optical LMRs which depend on the refractive index of the media surrounding this layer. In this section, sensors prepared with different metal oxides that work with distinct transduction principles are presented.

**Figure 22:**
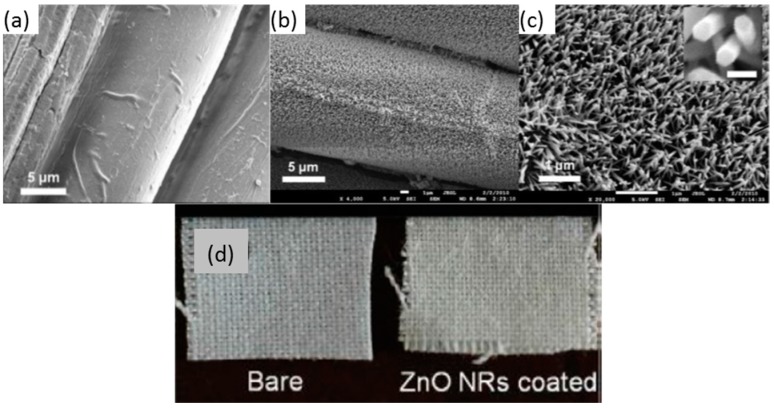
Cotton fibres (**a**) before and (**b**) after the ZnO deposition; (**c**) detailed of the coating structure formed by hexagonal shaped columns (see image inside); (**d**) fabric with no ZnO and another coated one. Reprinted from [102] and used with permission from Elsevier.

Zinc oxide (ZnO) is a relevant material due to its electronic features, chemical adsorption and the different ways available for its deposition and its adherence to distinct substrates. This oxide is a n—type semiconductor whose band gap energy is 3.37 eV [101]: gaseous O_2_ gets adsorbed into the metallic oxide, producing oxygen ions by taking electrons form the conduction band, which results in a reduction of the material conductivity. The phenomenon is also known as the modulation of the depletion layer by oxygen. On the contrary, some VOCs, such as ethanol, act as a reducing agent that releases back electrons to the conducting band, lowering the electrical resistivity. This reaction is also produced by H_2_, another relevant gas to be detected: in the case of this gas, the mechanism can be catalysed by thin a film of Pt or Pd. Radiofrequency sputtering has been used to deposit this oxide onto substrates of different nature, for instance, cotton fabric, taking advantage of its adherence [102]. The resulting layer shows a 1 micron thickness and it is not observable with naked eyed, although the cotton fibres are clearly coated at a nanometric scale (see Figure 22). ZnO has been also deposited on optical fibre by aqueous chemical growth as an alternative to sputtering [103]: a coating formed by nanorods was fixed on a LPG to produce spectral shifts in presence of ethanol vapours. The resulting thin film showed a thickness about just 100 nm, which gives an idea of the high sensitivity of this material. When illuminated with UV light, variations in a luminescent emission located at 390 nm were also registered when the sensor was exposed to the VOC (working in every case at room temperature). ZnO can be combined with other oxides such as SnO_2_ to prepare nanofibers sensitive to ethanol [104].

**Figure 23:**
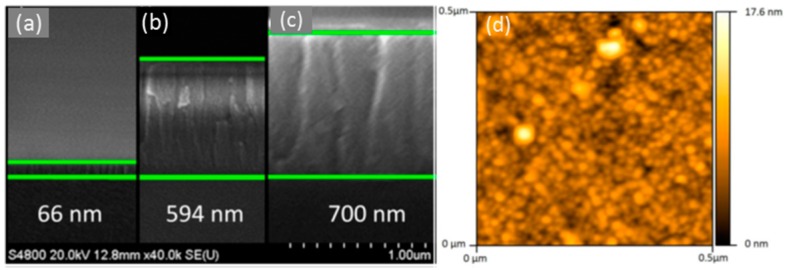
Thickness of sputtered SnO_2_ thin layers depending on the deposition time: (**a**) 30 s, (**b**) 3 min and (**c**) 4 min (inset, the measured thickness); (**d**) AFM image of coating (**b**), which shows a RMS roughness of 1.5 nm. Reprinted from [105] with the permission from Elsevier.

Tin dioxide (SnO_2_) is a widely used metal oxide in electronic sensors for VOCs and moisture measuring. As it discussed previously, these devices required the material to be heated up to promote the oxidation—reduction on its surface, which is not suitable for certain environments. This material shows a relatively high refractive index: this property has been employed to induce LMRs by depositing thin layers of the oxide around optical fibre segments where the cladding has been removed. The technique followed is sputtering, so that the final thickness of the layer can be controlled mainly by the deposition time, among other factors (see Figure 23): in any case, the final width is below 1 micron to ensure the transduction. 

The resulting coating induces lossy cladding modes which are affected by the refractive index of the layer itself and the environment: these changes produce a registrable spectral shift to characterize the sensor. Optical fibre sensors have been reported to detect and measure relative humidity based on LMRs [105,106]; interferometers prepared with photonic crystal fibres were also prepared with SnO_2_ with this aim [107]. Recent works are reporting its potential use for VOCs and gases detection. 

**Figure 24:**
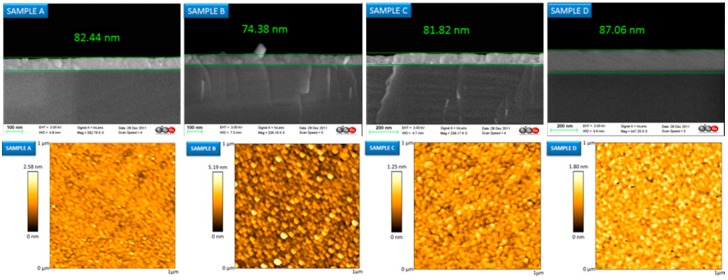
SEM images from the sputtered ITO samples with annealing under vacuum (sample A), nitrogen (sample B), atmosphere (sample C) and no annealing (sample D); on the second row, AFM images for the similar thermal treatments. Reprinted from [111] with permission from Elsevier.

Indium tin oxide (ITO) was one of the first oxides used to prepare sensing devices. This conducting material shares some properties with others but, due to its refractive index, it also induces LMRs. The first studies followed the dip coating procedure to deposit the oxide using a sol gel based mixture: the thickness of the final thin layer was optimized to 170 nm [108]. LMRs are affected by refractive index changes, so, taking that into consideration, coatings sensitive to different materials can be deposited onto the ITO thin layer to implement sensing devices: in this manner, Layer-by-Layer nanostructures [108] were used to develop humidity sensors. Thanks to the chemical nature of the oxide, it can be also deposited by sputtering, which shortens the construction process [109]. Other compounds can be coated around the sputtered ITO film: it is the case of polyvinylidene (PVdF), a conducting polymer that is sandwiched between two ITO layers to get a tuneable optical filter by electric signals: it can be used for electric field sensing too [110]. Another relevant property of ITO coatings is that the morphology can be adjusted not only by the deposition time but also by a post thermal treatment under different conditions. This effect was studied by preparing four different ITO coated surfaces [111]. After the metal oxide sputtering, they were annealed at 500 °C for 4 h under different conditions: vacuum, nitrogen, atmosphere and the forth one was not treated. As a result, the fourth coating was amorphous and show the poorest performance; the morphology of the other three ones is crystalline like, which makes easier the interaction with the environment (see Figure 24 for more details). It is important to remark that refractometers exhibiting giant sensitivity have been prepared by sputtering ITO on D shaped optical fibres [112]: they show a great potentiality in many sensing applications.

**Figure 25:**
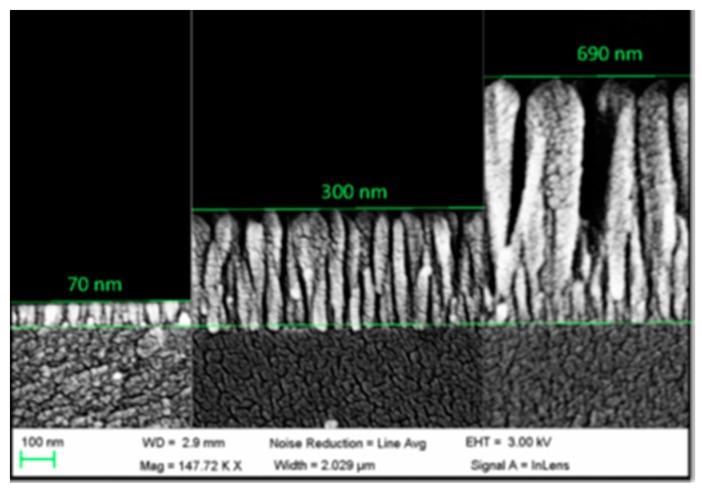
SEM images from In_2_O_3_ films with different deposition times: 30 s (70 nm), 150 s (300 nm) and 5 min (690 s). The resulting coating is homogeneous and it is formed by compact nanorods. Reprinted [116] from with permission from Springer.

It has been found that metal oxides with a high refractive index produce LMR for thinner thicknesses and, in some cases, dual peak resonances are induced: they can be referenced between them, which makes the system more robust against undesired fluctuations. More specifically, indium oxide (In_2_O_3_) has been used to prepare optical fibre refractometers based on LMRs: compared to other oxides such as ITO, it shows a higher refractive index and not only it produces a double—peak resonance, but also the sensitivity to external refractive index changes is improved by a factor of two [113]. This metal oxide was firstly deposited by dip coating technique in a way the final thickness depended on the number of immersion into the solution were it was dissolved [114]. In order to make the device more selective, different coatings can be deposited onto the In_2_O_3_ one: a humidity sensor was implemented by growing a layer-by-layer hydrophilic structure, so that relative humidity determined its refractive index [115]. The metal oxide can be also deposited by sputtering in a faster way if compared with dip coating: it has been observed that coatings below 100 nm yield to higher sensitivities to changes in environment refractive index. The layer morphology is compact but also rod-like (see Figure 25). A sensor prepared this manner was successfully evaluated for monitoring the aging of gear box oil as a function of its refractive index change [116].

Titania has been also employed to prepare optical fibre sensors. As an alternative to sputtering and dip coating techniques, this oxide can be deposited following a layer-by-layer method. Taking advantage of its refractive index, LMRs were induced for a 500 nm thick coating around an optical fibre: the final device is a refractometer based on spectral shift [117]. A FBG structure was prepared with this oxide, combining it with Al_x_O_y_: alternating layers were deposited onto a cleaved ended fibre by physical vapor deposition. The last layer of the nanostack can be functionalized with a sensing material in a way that variations in its refractive index would shift the spectral notch of the grating [118].

### 4.6. Carbon Nano Tubes (CNTs)

Developed for the first time by Iijima on the early 90s [119,120], CNTs are considered a key nanomaterial, and it encouraged many researches to focus on nanotechnology. Other materials based on carbon, such as graphene, have also been studied for the development of optical fibre sensors [121]. The unique features of CNT are a consequence of their chemical structure: high electrical conductivity, mechanical robustness, near perfect black body characteristics and, regarding to the sensing field, they show a high surface/volume ratio (between 100—1800 m^2^/g). Roughly, there are two types of CNT: single walled (SWCNT), which have a hollow nanostructure where the carbon atoms are organized in single rolled tubes and multi walled (MWCNT), where several single tubes with different diameters are grouped in concentrically. The dimensions of CNTs—both diameter and tube length—are always on the nanometric scale; these parameters define properties such as the reactivity or the optical absorbance. 

They are normally used separately, although some authors proposed to employ them jointly [122]. Both types of CNT have been employed to elaborate optical fibre sensors. SWCNT have been used to implement VOCs sensors: following Langmuir Blodgett technique, several layers of the sensing material were deposited at the end of a standard optical fibre to build up a Fabry Perot interferometer [123,124]. Due to the electrical nature of CNT, electrons from VOCs such as toluene react with the carbon atoms of the tubes, producing changes in the refractive index and so, a measurable variation in the reflected optical power. Despite of the interaction surface available, this type of deposition for the SWCNT offers a low reproducibility when preparing sensors. Another work proposes incorporating the SWCNT into a cadmium arachidate (CdA) matrix to overcome this issue: following a similar reflection architecture, the resulting device was able to individually distinguish between ethanol, ethyl acetate and toluene vapours [125]. Other gases, such hydrogen, have been detected by optical fibre sensors prepared with SWCNTs: thanks to their molecular morphology, the tubes adsorb the gas molecules, which induces changes in the dielectric constant and thickness of the sensing material [126]. Sensors based on SWCNTs to detect toluene vapours have been also reported [127].

The functionalization of MWCNTs has yielded optical fibre sensors that combine this sensing material with other transduction principles. Luminescent compounds can be attach to MWCTs in a way their emission is modified by the entity to be measured: it is the case of fluorescein molecules, which can be linked to the nanotubes walls by covalent bounds [128]; in order to reduce self-quenching, as well as increasing the water solubility, polyether spacers were included between the tubes and the dye. An extrinsic optical sensor was developed with this material: it was able to measure pH changes in a 4.5–8 range. 

**Figure 26:**
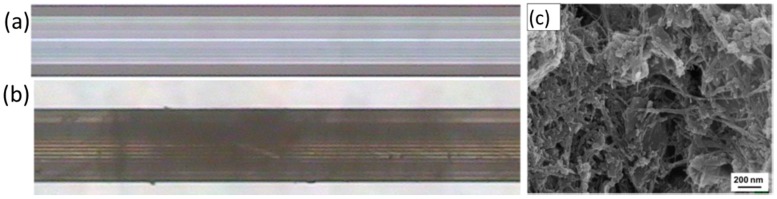
Images from an optical microscope (**a**) of the PCF before and (**b**) after the CNT deposition; (**c**) SEM capture of the CNT doped with copper nanoparticles coat deposited on optical fibre. Reprinted from [130,131] with the permission of Elsevier.

As it was mentioned before, MWCNTs also show relevant optical properties: when they are illuminated, their structure changes in terms of bending and stretching, but, what is more important, these variations are reversible. Considering their photo-elastic nature, MWCNTs have been coated by chemical vapor deposition onto fibre Bragg gratings: the resulting layer was just 50 nm thin and about 60 microns long [129]. Depending on the intensity and wavelength at which the coating is illuminated, the mechanical strain on the grating is different, which is transduced to a shift in the reflected Bragg wavelength. Not only a UV or IR radiation sensor can be implemented, but also an optic modulator or filter. CNT can be also combined with PCF to get a refractometer based on interferometry. Taking advantage of the one dimensional structure, the nanotubes can be dissolved and sprayed towards a silica surface because they get strongly bonded [130]. When working with a PCF interferometer, the evanescent field is enhanced, so there is a more remarkable interaction with the sensing coating, in this case, the sprayed CNT (see Figure 26a,b): in this way, the sensitivity to variations in the environment refractive index is increased. Finally, it is also possible to deposit MWCNT onto metallic surfaces: in the case of optical fibre, a metallic thin layer can be coated to induce SPRs, so that the nanotubes would make it sensitive in a manner the wavelength location is shifted in presence of the target analyte; to get selectivity to nitrates, a study proposed to include copper nanoparticles into the tubes. This metal acts as a catalyst with nitrates to produce ammonium ion, in whose reaction the CNT electrons are required. The resulting material (see Figure 26c), is dip coated onto the metallic layer deposited on the fibre, and produces shifts in the SPR induced by the metal [131].

### 4.7. Nanowires

There are different concepts related to nanowires in the literature, where two of them are highlighted: stretched optical fibres with a diameter in the nanometrical range and nanostructures with rod shape that show certain optical properties. The first type of nanowires is prepared by the flame—brushing method or by applying an electrical arch: in this manner, the fibre is heated and pulled to reduce its diameter down to the nanometric range. This procedure can be also applied to other materials such as semiconductors with a crystalline structure or metals. A precise control of the both the mechanical pulling and fibre heating is required to ensure an acceptable reproducibility. To be considered as a nanowire, the final diameter along the stretched section has to be lower than the working wavelength [132]. The resulting device is a 1D nanostructure that exhibits a mechanical robustness typically better than stretched fibres with wider cores and, moreover, it is observable by an optical microscope, which makes easier its manipulation to prepare, for example, nanorings or nanoloops [133]. The evanescent field along the stretched segment is highly increased, which makes it very sensitive to the changes of the surrounding refractive index changes: therefore, many optical fibre nanowires are used as refractometers. The theoretical background is explained by the Maxwell equations and by the H and E components of the propagating signal: there is a clear relationship between the final sensitivity and the stretched fibre diameter, which can be studied by finite element method [134]. The common configuration of this kind of devices is transmission or a Mach Zehnder interferometer: for the second one, a non-functionalized nanowire is used as reference in one of the interferometer arms, whereas another with a sensing coating is connected to the other wire [135]. There are other configurations such as the hybrid one, which consists on a nanowire fused at the end of a cleaved ended fibre [136]: this architecture combines the evanescent field increased interaction with the mechanical properties of reflection sensors. To highlight the versatility of nanowires, they can be combined with MOFs, which allows the resulting device polarization properties to be controlled [137].

Once the fibre is tapered, its surface is functionalized to provide the sensor with the required sensitivity to the target analyte. A simple procedure to do it is silanization: it consists of just dipping the stretched segment into a silanizing solution (for example, methoxysilane), which makes the fibre’s cladding non-sensitive to refractive index changes produced by relative humidity but, for example, to certain apolar gases. An optimized study about the effect of the stretched diameter describes sensors with just of a few millimetres of nanowire and a 800 nm core diameter are enough to detect propane leakages [138]. Moreover, some authors presented a reflection sensor based on nanowires that included a structure to improve its mechanical robustness: the resulting devices can therefore be functionalized to sense different parameters [136]. 

**Figure 27:**
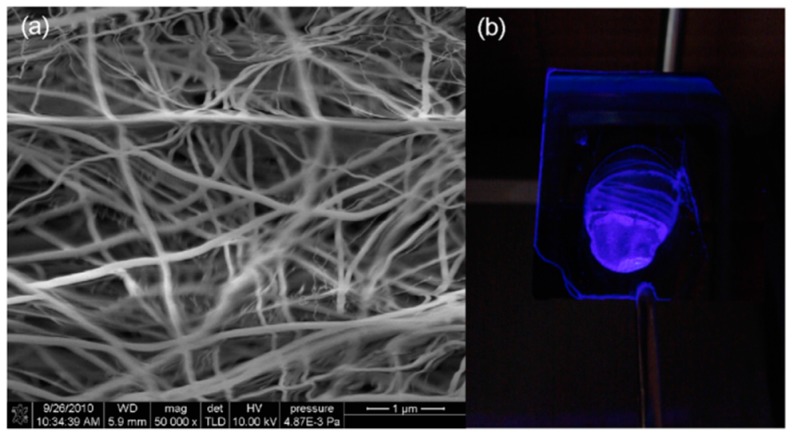
(**a**) SEM image of the silica nanowires functionalized with cryptophane-A; (**b**) luminescent emission of the nanowires when illuminated with UV radiation. Reprinted from [142] with permission from Elsevier.

Another different concept of nanowires is related to the synthesis of structures with nano rods geometrics that can conform arrays around or at the end of optical fibre [139] or to fill, for example, the air holes of MOFs [140]: actually, SPRs can be induced in this way with silver nanowires filling randomly PCFs air holes [141]. Furthermore, nanowires can be used to prepare extrinsic optical fibre sensors. Silicon monoxide can be exposed to high temperatures to obtain, after a gradual cooling rate, silicon nanowires: a sensor to detect methane was reported by adding to the nanowires cryptophane-A [142], yielding to a luminescent sensing membrane (see Figure 27) whose emission is quenched by the target gas. 

**Figure 28:**
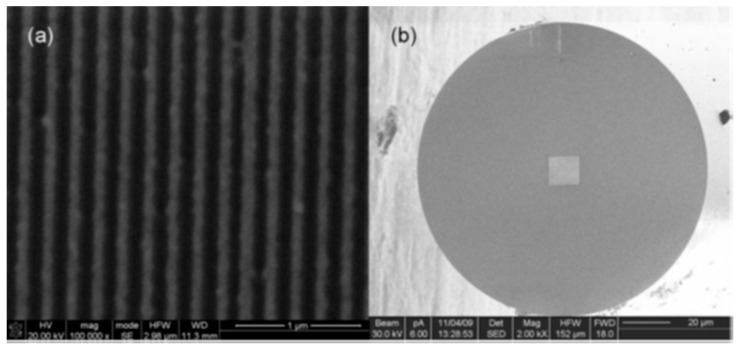
(**a**) Detail of the gold grid fabricated on the tip of a SMF; (**b**) SEM image of the sensor head: the square grid is appreciable at the centre of the fibre. Reprinted from [143].

Finally, the term nanowire can also refer to elements of a grid or pattern with certain optical properties that are affected by variations in electric or magnetic field. The resulting deposited material looks like parallel lines, so that is the reason why they are named nano wires. The materials employed to prepare the grid are transition metals such as platinum or gold (see Figure 28) [143]. To get a better sensitivity, the fibre can be D shaped and, onto the flat surface, layers of metals following a pattern are deposited: the pattern is obtained by using a laser beam through a mask [144]. The device is based on SPR induced by the metallic layers, and they are red shifted by the exposure to UV radiation.

### 4.8. Molecularly Imprinted Polymers (MIPs)

**Figure 29:**
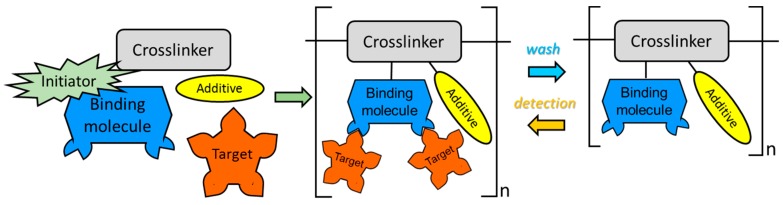
Brief synthesis and reaction processes for a MIP, showing the most relevant molecules involved.

This kind of materials is molecularly designed to be sensitive to a certain target molecule. Although they are not strictly nanostructured, the possibility of designing them at a molecular level justifies their description in this review. MIPs were developed as an alternative to biological molecules with a high selectivity, which is the case of enzymes or anti bodies: these molecules have to operate typically under physiological conditions to prevent their degradation, which sometimes makes difficult their use for sensor development. On the contrary, MIPs are synthetic molecules that can be deposited and used with different working conditions. To stablish an analogy, a MIP acts as a lock which can be open only by a certain target molecule, so that the selectivity is very high: a properly designed MIP could distinguish even between isomers, for instance, glucose and fructose. In order to synthesize a MIP, different chemical reagents are mixed in a solvent (typically an organic one, although water has been also used) together with the target molecule, which acts as a template. The reagents are in most cases these ones: a monomer which constitutes the backbone of the resulting MIP; other monomers with functional groups that guarantee the reaction with the target molecule; chemical dyes that will be also included in the final reagent whose optical properties (such as luminescence) vary depending on the presence of the template; a cross-linker that make possible the synthesis of the new polymer and a starter agent. During the reaction, the MIP morphology is determined by the target molecule in a way its functional groups are linked with it by weak forces (hydrogen bonding, Van der Waals forces, among others): once the chemical reaction is complete, the template can be removed by a solvent and linked again to the MIP reversely. Figure 29 shows the process. The selection of the proper reagents determines the final features of the MIP, so it is a critical step: the functional groups, the binding energy to the template or the hydrophobic behaviour are just some critical parameters that should be considered. Actually, some authors have already computationally design MIPs to get the best results [145].

The synergy between MIPs and optical fibre is clear due to the high selectivity and all the very well-known features of the substrate: the most critical step for their combination is the deposition of the sensing material on the fibre due to its small dimensions. Therefore, most works report the use of wide core fibres, such as PCS or POF, which offer an active area where the MIP can be effectively deposited. As it will be explained in the following paragraphs, some authors prefer the polymerization to take place onto the fibre, whereas other deposit the sensing polymer once the reaction is over.In the case one of the monomers included during the polymerization process is, for instance, luminescent, optical fibre can be used to directly measure the changes induced by the presence of the target molecule: this is the case of sensors prepare to detect cocaine with a high selectivity [145,146]. For these devices, the fibre is initially treated to have an anchoring layer where the polymerization will occur: thereafter, the fibre is inserted into a vial where all the reagents are present; once the process is over, the fibre is removed from the vial and the sensor is ready to be used. There are other approaches in which the fibre is immersed into a solution where the polymerization reaction has been completed, so that the MIPs is already dissolved: in this manner, the fibre does not have to be pre-treated and the sensitive film is deposited by dip coating [147,148]; however, depending on the MIP, the resulting sensor is to be used at once because the template cannot be removed from the polymer.

Following the previous idea of depositing the MIP molecules, some authors propose to etch the optical fibre (on its top or around its coating) to create holes where the sensing molecules are to be located. In this manner, an array of fibres with different MIPs can be used to form a bundle and in this manner, a multi parameter device is obtained (also taking advantage of the multiplexing feature of the fibre). Polymers with spherical shapes are easier to locate on the etched surface: fluorescent MIPs have been developed this way to create antibiotic sensors based on the luminescent emission of the polymer [149].

Other works report the combination of SPRs and MIPs: the fibre is firstly coated with a metallic thin layer (gold in most cases due to its plasmon resonance spectral location) and then, the polymerization mixture is drop coated onto it, taking place the MIP synthesis after a curing. The resulting sensor varies the SPR location in presence of the target molecule, which is an alternative to luminescent emission based schemes: actually, the fibre can be D shaped to enhance the SPR [150] or the MIP can be located onto polished fibres that form an optical coupler [151]. These devices have been developed to control oil degradation in transformers, detecting the presence of dibenzyl disulphide (DBDS) and furfural. 

**Figure 30:**
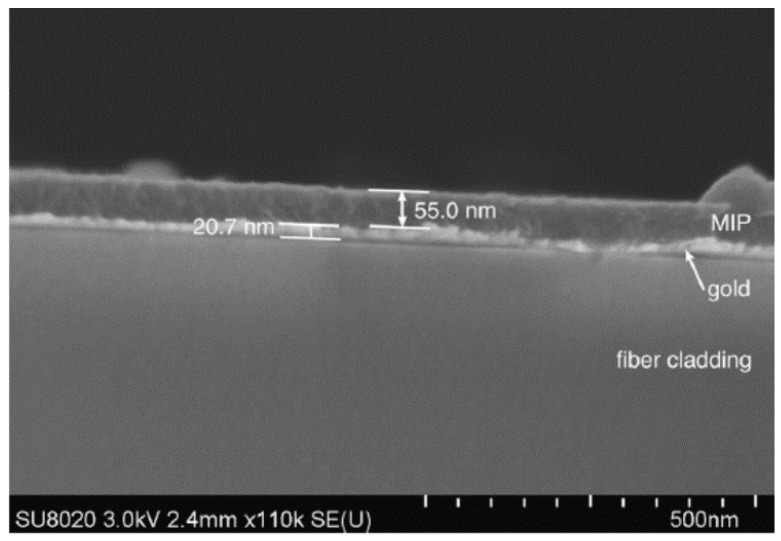
SEM image for a MIP coated onto an optical fibre by electropolymerization. Reprinted from [152] with permission from Elsevier.

The polymerization is currently being adapted to the optical fibre in a way it plays an active role during the reaction: as before, a metallic coating is sputtered on to the fibre to generate a SPR, but also, to be used as an electrode. In the case a conducting monomer is used as backbone (polypyrrole) in the reaction, electro-polimerization can be used to induce the MIP synthesis, ensuring that it is properly attached onto the substrate. The three layers involved (fibre cladding, gold thin coating and MIP) are shown in Figure 30: even with a thickness below 100 nm, a SPR spectral shift of 2 pm per formaldehyde ppm has been reported [152]. 

### 4.9. Layer-By-Layer (LbL) Nanostructures

**Figure 31:**
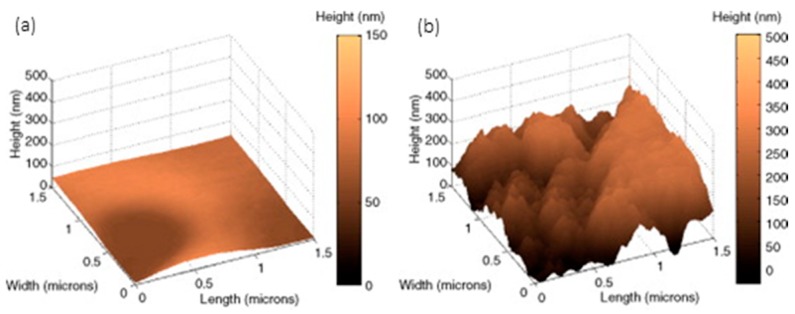
(**a**) AFM image from a [PAH+HTTPS/PAA] coating (left hand side image) and one with a hydrophilic coating deposited below (right hand side image); (**b**) the second one shows a higher roughness that enhances the sensitivity of the sensor. Reprinted from [155] with permission from Elsevier.

**Figure 32:**
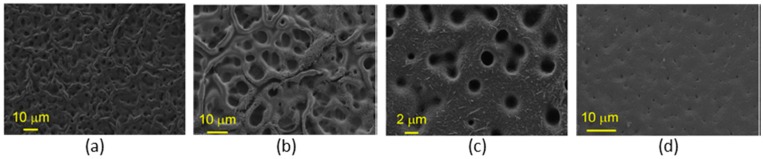
SEM images from [PAH/PAA] nanostructures that have been embedded with organometallic materials that share the [(C_6_F_5_)_2_Ag_2_Au_2_L_2_]_n_ backbone, where L is a ligand molecule. Depending on the ligand, morphologies with different porous size are obtained: (**a**) 1/2 diphenylacetylene, (**b**) ammonia, (**c**) pyridine and (**d**) 2,2’-bipyridine. Reprinted from [157] with permission from Elsevier.

The small dimensions of optical fibre sometimes make it difficult to deposit coatings, but following LbL, this problem is overcome for both reflection and transmission configuration. The first sensors developed were for the first architecture to prepare nanointerferometers: LbL versatility allows polymers of different types to be used, and as a consequence, different morphologies to be adjusted at a nanometric level. The resulting cavities grown at the end of cleaved ended fibre are commonly prepared to show a thickness below 1 micron to get an interferometric response: if the coating is sensitive to humidity variations in a way its refractive index is modified, the interferometric signal follows this change. Taking this effect into consideration, a humidity sensor was reported by combining a gold colloid mixed with poly (diallyldimethylammonium chloride) (PDDA) and poly (sodium 4-styrene sulfonate) (PSS); the cavity thickness was estimated to be 310 nm, and was one of the first optical fibre sensors prepared by LbL [153]. The hydrophilic nature of most of LbL structures helps materials dissolved in water to be diffused into the supporting matrix: what is more, a wide range of compounds can be deposited at the same time with the polymers involved, or they can even act as a charged molecule. Prussian Blue, a chemical dye, has been deposited with a poly(allylamine hydrocholoride) (PAH) and poly(acrylic acid sodium salt) (PAA) by mixing it with the first reagent to measure H_2_O_2_: a [PAH/PAA]_2_ final coating was found to prevent leaks [154]. Sensors to measure pH have been also obtained by including indicators to one of the polymeric solutions: it is the case of HPTS, which is deposited together with PAH using PAA as polycation. Variations in absorbance spectrum were used to characterize the sensor response. Furthermore, it was observed that the performance of the sensor could be improved by firstly depositing layer pairs with a remarked hydrophilic behaviour: AFM images confirm this phenomenon (see Figure 31) together with the obtained results [155]. It has been also observed that the some polymers, specifically, the combination of PAH and PAA, are sensitive to pH variations, producing a reversible swelling effect that can be registered by the interferometric response in this type of reflection configurations [156]. Moreover, [PAH/PAA] nanocavities have been embedded with sensing materials to obtain VOCs sensors: the initial morphology showed a high porosity that was altered by the organometallic compounds used to prepared the devices (see Figure 32) [157]. 

**Figure 33:**
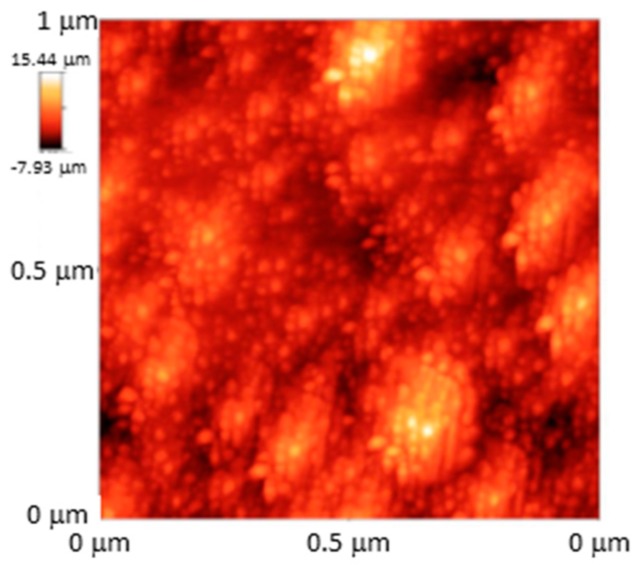
AFM image for the LbL nano coating deposited onto a LPG and embedded with SiO2 nano spheres. Reprinted from [160].

The sensitivity of devices based in transmission configuration can be functionalized by LbL: as an important example, LPGs are sensitive to the external refractive index, in a way the resonance wavelength is shifted by this parameter. It was theoretically proposed that thin layers could improve this sensitivity significantly, but to achieve that, a precise control of the thickness was required [158]. In this background, LbL proved to be an optical technique to deposit thin films with a resolution at a nanometric level: firstly, the hypothesis was confirmed by a just few nanometers thick mono layer. The wavelength shift was not only affected by the refractive index of the coating but also by its thickness: in this manner, 25-layer pairs of [PAH/PAA] were deposited onto a LPG to get a pH sensor, yielding to a 200 nm thick coating. The resulting nanocoating is swelled by pH fluctuations, which is transduced in terms of the spectral shift [159]. To get selectivity for a certain analyte, the nanodeposition can be doped with a sensing material: it is the case of SiO_2_ nanospheres, which make the total refractive index of the coating depend on environmental humidity, forcing the shift of the LPG resonant peak [160]. AFM images confirm that the resulting coating shows a grain-like morphology that easies the interaction with water molecules (see Figure 33).

**Figure 34:**
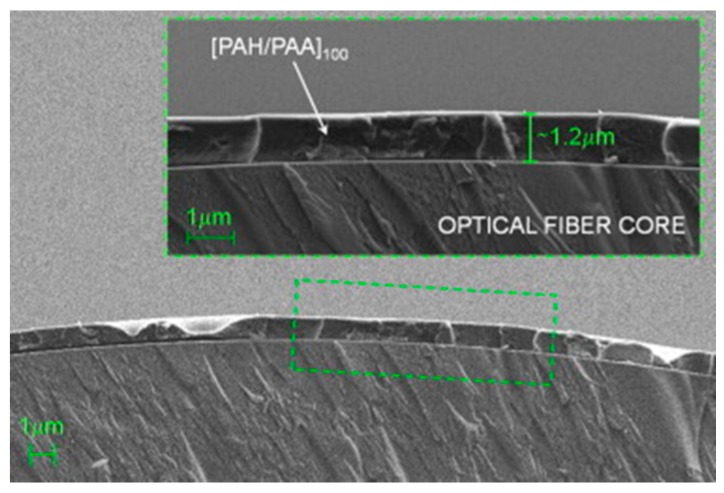
SEM image of a [PAH/PAA]_100_ coating onto a PCS fibre. Inset, a detail of the thin layer. Reprinted from [162] with permission from Elsevier.

One of the most relevant contributions from LbL to optical fibre sensors is the deposition of materials that induce LMR. Firstly, these resonances were coupled by metallic materials, but it was found that polymeric coatings with a high refractive index are also able to induce these resonances: moreover, it was discovered that transmission sensors based on this transduction show a better sensitivity when compared to LPG sensors [161]. The use of PCS fibres allows the cladding to be easily removed, so that LbL nanostructures can be deposited to operate as transmission sensors. In the case of using coatings that show a swelling effect depending on pH, the coating thickness is modified, shifting the LMR spectral location: as an example, [PAH/PAA] layer pairs, with an averaged thickness of 12 nm, were grown onto PCS fibre to obtain a compact coating sensitive to pH (see Figure 34) [162]. 

LbL coatings can be also embedded with sensing materials to induce SPRs as well as LMRs, so that a self-referenced device is implemented. It is well known that nanoparticles can be used to dope coatings in a way the resulting refractive index is defined by its concentration. Silver nanoparticles were loaded into a [PAH/PAA] nanostructured for this propose. Two methods are reported: on one hand, the layer pairs are deposited and then immersed into a solution where the nanoparticles are dissolved; on the other hand, the Ag nanoparticles are mixed with PAA and deposited along the LbL growing. The second procedure yields to a more uniform distribution of nanoparticles, and so, a better sensitivity. Results were evaluated and confirmed by relative humidity measurements [163]. 

**Figure 35:**
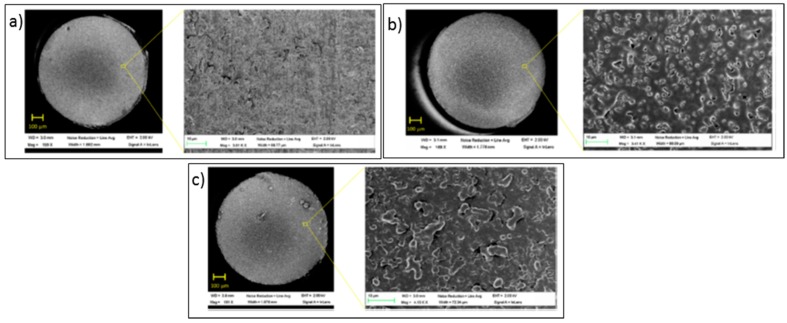
SEM images obtained from sensor heads deposited with Pt-TFPP with 3 different cationic polymers: (**a**) PDDA, (**b**) PEI and (**c**) PAH. A general view of the fibre and a detailed area of the coating is displayed for each coating. Reprinted from [165] with permission from Elsevier.

To conclude this section as well as to highlight the versatility of LbL nanostructures, luminescent dyes have been also deposited by this technique in a reflection configuration. An oxygen sensitive metallic porphyrin (Pt-TFPP) was coated by preparing anionic micelles and combining it with PAH [164]. It was also found that among three different polymers (PDDA and PEI), the PAH prepared coating showed the highest roughness (21 nm RMS) and a porous morphology optimal for oxygen gas diffusion (see Figure 35) with a thickness of just 180 nm [165]. In order to minimize self-quenching of the luminescent molecules, structures with that included [PAH/PAA] pairs between the luminescent material layers were studied: not only a better sensitivity was registered but also the kinetics were improved. The resulting thickness was 147 nm and their roughness 45 nm RMS [166].

### 4.10. Electrospun Nano-Fibres

**Figure 36:**
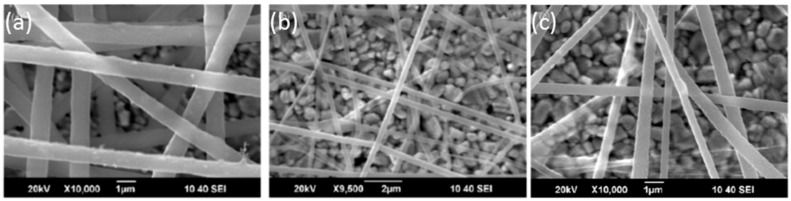
SEM images from electrospun nanofibres for PMMA concentrations in DMF solutions of (**a**) 0.32 mg/mL and (**b**) 0.18 mg/mL; (**c**) final fibres when the PMMA/PANI complex is synthetized. Reprinted from [167] with permission from Elsevier.

The nanomembranes obtained by electrospinning exhibit a high surface/volume ratio, which enhances the interaction between the sensing material and the target analyte. Polymers are dissolved to obtain the nanoweb structure. As an example, poly(methyl methacrylate) (PMMA) has been used to detect organic vapours such as triethylamine (TEA) [167]: more specifically, the polymer is dissolved in dimethylformamide (DMF) and electrospun onto glass slides. The resulting matrix is polymerized with aniline to obtain polyaniline (PANI), which is sensitive to TEA: the nanofibres diameters ranged between 900 nm and 250 nm depending on PMMA concentration; once PANI polymerization is over, the final diameter is between 400 and 600 nm (see Figure 36). The sensing matrix is finally deposited onto an electrode because the electrical structure of PANI varies reversely in presence of TEA: a linear relationship between the electrode electrical signal and the VOC concentration was found up to 500 ppm. 

**Figure 37:**
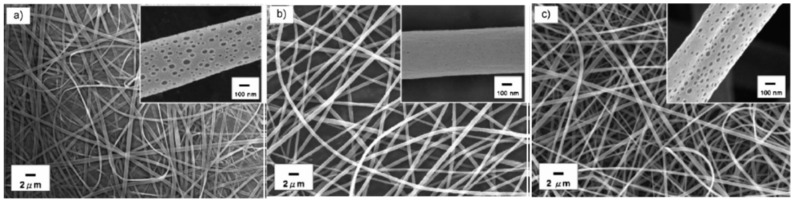
SEM Images that show the different nanofibre morphologies when (**a**) CH_2_Cl_2_, (**b**) CB and (**c**) CHCl_3_ are used as solvents. Reprinted from [168] with the permission of John Wiley and Sons.

The optical absorption spectra of the previous material was also affected by TEA, so that it could be used for an optical fibre sensor. In this manner, looking forward optical detection luminescent materials have been also deposited by electrospinning. An strategy consists of binding a fluorescent indicator such as pyrene methanol (PM) to a polymer chain, for example, poly(acrylic acid) (PAA) [33]. The indicator is linked to the polymer by covalent attachment, forming a material whose fluorescence is quenched by the presence of metal ions (Fe^3+^ and Hg^2+^). Another luminescent copolymer has been successfully deposited to follow pH variations [168]: in this case, the importance of the solvent is studied because it defines the final morphology of the electrospun nanofibres (see Figure 37): dichloromethane (CH_2_Cl_2_), chlorobenzene (CB) and chloroform (CH_3_Cl) were used; the best results were obtained with the first solvent. The emission spectrum of the polymer is red shifted when pH is decreased, which can be used to characterize the sensor.

As another example of the versatility of electrospinning, oxygen-sensitive porphyrins have been successfully deposited following this method: platinum tetra(pentafluorophenyl) porphyrin (PtTFPP) shows a luminescent emission that is reversibly quenched in the presence of oxygen, and it has been used to obtain non-woven sensing matrices [36]. The porphyrin is dissolved together with polystyrene (PS) in 2-butanone with a surfactant: the resulting coating showed a 620 nm thickness and the average fibre diameter was around 100 nm. Other luminescent electrospun nanofibres have been reported for the detection of nitrobenzene [169]; moreover, metallic oxides such as ZnO can be also used to obtain nanofibres by this technique [101] as well as luminescent hybrid composites combining polymers and gold nanoparticles [170].

**Figure 38:**
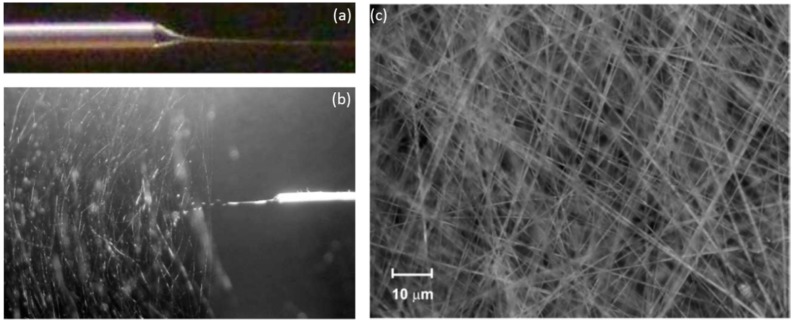
(**a**) Detail of the needle where the jet emerges and (**b**) the nanofibre formation, describing a helix path; (**c**) SEM image of the nanoweb once it is deposited onto a glass slide. Images reprinted from [171] with the permission of IEEE.

Poly(vinylidene fluoride) (PVdF) has been electrospun and used as sensing material for relative humidity monitoring [171]. The procedure is similar as in the previous examples: the polymer is dissolved in a mixture of acetone and N,N-dimethylformamide and then, it is injected into a syringe and exposed to an 18.5 kV electric potential differential. The optical fibre was located close to the grounded electrode, and it was rotating at a constant speed of 120 rpm during the process. The final coating had a 6 microns thickness, whereas the diameter of the nanofibres were between 100 and 700 nm: details of the electrospinning process can be observed in Figure 38. The nanofibres were deposited around a HCF to prepare a transmission sensor: it was illuminated with a laser at 1310 nm, registering a decrease in the transmitted signal as the relative humidity increases. This effect is produced by the interaction between the coating and the evanescent field of the light traveling through the area where the mat is deposited. The sensor was also validated for human breath monitoring, taking advantage of the fast response produced by the high surface/volume ratio of the nanoweb. 

**Figure 39:**
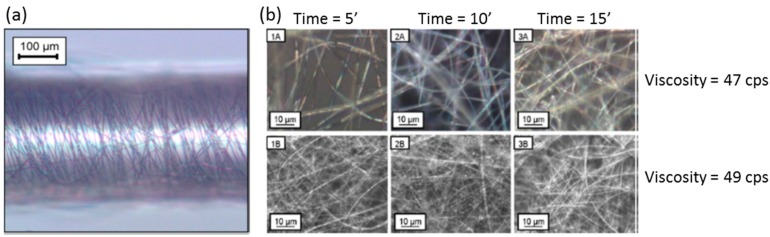
(**a**) Microscope image of an optical fibre core coated with the PAA electrospun fibres; (**b**) SEM images for the nano webs obtained with the different electrospun conditions (detailed inset). Reprinted from [37] with the permission of Elsevier.

There are commercially available polymers that are already dissolved, which can make easier the process: poly(acrylic acid) (PAA), provided in aqueous solution, can be directly used in the electrospinning set-up; this material swells with humidity due to its hydrogel nature: this property has been used to develop an optical fibre humidity sensor [37]. The fibre used is PCS, and the nanoweb is coated around a region where the cladding has been removed; it is again located between the electrodes and it is kept rotating at 30 rpm. The importance of the PAA solution viscosity as well as the electrospinning time is found to be critical for the sensor features: the more viscose is the PAA solution, the smaller is the averaged diameter of the nanofibre and therefore, the higher is the density of the mat deposited; on the other hand, the sensitivity is directly affected by the thickness of the coating, which depends on the deposition time (the longer it is, the thicker is the mat). A summary of the different nanofibres obtained with distinct viscosities and deposition times is displayed in Figure 39. There is a trade-off between all the parameters to get the best sensitivity: the optimal nanofibres should be as thin as possible, and the thickness of the coating as low as possible; for this study, the best results were obtained with the lowest deposition times (5 min) independently of the PAA concentration.

## 5. Conclusions

It is evident that there is a synergy between optical fibres and micro/nanostructured materials to develop chemical sensors and biosensors. The construction and deposition methods are critical when defining the morphology of the sensing coating and therefore, its features. The sensing architecture, reflection or transmission, has to be chosen depending on the requirements of the application, and depending on that, the construction technique as well as the materials to be used are to be selected. Regarding the construction techniques, although dip coating is easy to perform, its low reproducibility, specifically for reflection sensors, makes the other techniques preferable. LbL allows not only polyelectrolytes to be deposited, but also reagents from different types such as biomolecules, organometallic materials or even metal oxides. Its versatility and the possibility of applying it for both transmission and reflection configurations is extending its use for optical fibre sensor development. Talking about sputtering, it can also be used for both configurations and it guarantees to prepare similar devices under the same conditions. Initially it was only employed to metallic and metal oxides, but now, the use of RF signals allows materials of different types to be deposited, which will encourage many researches to follow this method. Finally, nanofibres can be electrospun to prepare transmission devices with a relative simple setup easy to adapt to optical fibre sensors. Although there are not many sensors developed this way, this construction technique is very promising.

New materials allow nanostructures to be deposited onto the fibre which enhance the interaction with the analyte: they show a great aspect ratio, which easies the diffusion of the target molecules. In the case of plastic matrices and sol gel, the supporting matrix morphology is easy to control, although they are restricted to transmission configuration to guarantee repetitive sensors. Carbon-based materials such as carbon nanotubes are also very promising which have been reinforced by the recent results obtained with graphene in the sensors field. Regarding the sensing material rather than the supporting matrix, MIPs offer a great selectivity and sensitivity: their main inconvenience is the synthesis of the polymers and their attachment to a supporting matrix that does not alter their sensing properties. The materials that currently show a great applicability are LbL nanostructures and sputtered metal oxides: the first ones are very versatile in terms of the compounds that can be deposited, whereas the second ensures a high reproducibility when preparing the sensors; moreover, both are applicable to reflection and transmission configurations. To conclude with nanostructured materials, electrospun nanofibres can be aligned to produce certain patterns that show a great porosity optimal, for instant, to detect gas or vapours. More materials are to be studied in order to obtain sensing layers in this way.

The benefits of optical fibre sensors based on this kind of materials are still to be explored, but the combination of this substrate and nanostructured materials give them a chance to cover niche applications where electronic sensors are not applicable. In any case, the results exposed in this review show a promising future that will be bring into reality in a medium term.
